# Programming virulent bacteriophages by developing a multiplex genome engineering method

**DOI:** 10.1128/mbio.03582-24

**Published:** 2025-05-23

**Authors:** Hailin Zhang, Ru Zhu, Zhaofei Wang, Ruoting He, Yuran Zhang, Ji Luan, Yaxian Yan, Youming Zhang, Hailong Wang

**Affiliations:** 1 State Key Laboratory of Microbial Technology, Institute of Microbial Technology, Helmholtz International Lab for Anti-infectives, Shandong University–Helmholtz Institute of Biotechnology, Shandong University 520252, Qingdao, Shandong, China; 2 School of Life Sciences, Jining Medical University 74496, Rizhao, Shandong, China; 3 School of Agriculture and Biology, Shanghai Jiao Tong University, Shanghai Key Laboratory of Veterinary Biotechnology 596493, Shanghai, China; 4 Rizhao Research Institute of Shandong University, Rizhao, Shandong, China; Freie Universitat Berlin, Berlin, Germany

**Keywords:** phage engineering, synthetic phages, genome engineering, recombineering, phage chassis

## Abstract

The use of virulent bacteriophages (phages) against pathogenic bacteria has recently attracted considerable interest. The limitations of naturally isolated phages have promoted the development of genome engineering methods to optimize their functions; however, engineering of virulent phage genomes in bacterial hosts remains challenging. Here, we describe a SMART (splitting, modifying, assembling, and rebooting) method for multiplex genome engineering of virulent phages by splitting the genome into multiple segments cloned and inserted into single-copy bacterial artificial chromosome vectors in *Escherichia coli* to overcome the toxicity of phage gene products, modifying multiple targeted loci in parallel through recombineering, assembling split segments into an intact genome, and rebooting the recombined phage in bacterial hosts. Using the SMART method, the 39.9-kb T7 *E. coli* phage genome was split into 10 segments, and 8 genomic loci were deleted in parallel to obtain a chassis phage with 10% genome reduction. The insertion capacity of the chassis phage genome and the expression levels of exogenous genes at different loci were evaluated. Finally, a synthetic T7 phage that efficiently lyses widely different bacteria, such as *E. coli*, *Staphylococcus aureus*, and *Streptococcus agalactiae*, was constructed by expressing heterologous lysins from *Staphylococcus* and *Streptococcus* phages. SMART will facilitate the programming and understanding of the genome structure and function of virulent phages.

**IMPORTANCE:**

Unlike temperate phages, which integrate into host genomes and allow time for genetic manipulation, lytic phages rapidly hijack the bacterial machinery and trigger host lysis within minutes, leaving an extremely narrow editing window. Furthermore, virulent phage genes usually encode toxic products that inhibit the growth of bacterial hosts. We developed a SMART (splitting, modifying, assembling, and rebooting) method for multiplex genome engineering of virulent phages. We deleted 3.9 kb sequences distributed across 8 sites in the 39.9 kb genome of the wild-type T7 *Escherichia coli* phage to construct a chassis phage. Synthetic T7 phages expressing heterologous lysin genes were constructed to efficiently lyse different bacteria, such as *E. coli*, *Staphylococcus aureus,* or *Streptococcus agalactiae*. The SMART method will facilitate targeted modifications of phage genomes for the creation of custom-designed phages with enhanced therapeutic efficacy, broader host specificity, and programmable behaviors.

## INTRODUCTION

Bacterial antibiotic resistance caused by the irrational use of antibiotics is becoming increasingly severe and poses a serious threat to both human and animal health (1, 2). New and effective means to combat antibiotic-resistant bacteria are urgently needed. However, the development of new antibiotics is proceeding slowly and fails to fully meet the needs of clinical treatment. Among the alternative solutions being explored, virulent bacteriophages (phages) are currently among the most promising candidates for development as therapeutic agents against antibiotic-resistant pathogenic bacteria (3–7). As viruses that infect bacteria, virulent phages strictly replicate themselves in bacterial hosts through the lytic lifecycle (8, 9). The stringent host specificity of phages necessitates the isolation of specific phages for each pathogenic bacterium, adding complexity and cost to clinical applications (10, 11). Furthermore, virulent phages for a considerable number of pathogenic bacteria have not been isolated. The ability to engineer phage genomes is central to the construction of artificial phages with improved lysis rates and spectra.

The desired mutations can be introduced into the target locus of the virulent phage genome via *in vivo* homologous recombination in infected bacterial cells. A method that relies on homologous recombination between a plasmid carrying mutations flanked with phage DNA (homology arms) and the phage genome has previously been developed (12). Although this method was optimized by introducing recombineering to improve the efficiency of homologous recombination and introducing CRISPR‒Cas counterselection to facilitate the isolation of the desired recombinant phages with the edited genome (13–16), it is restricted to limited genomic loci because many virulent phage genomic segments encode toxic products that inhibit the growth of the *E. coli* cloning hosts and the phage-infecting bacterial hosts (17). To overcome the difficulties in cloning toxic phage genomic segments, another method termed BRED (bacteriophage recombineering of electroporated DNA) was developed. In BRED, the PCR-amplified recombineering substrates do not need to be cloned and inserted into a plasmid but rather need to be directly electroporated together with the phage genomic DNA into the bacterial host cell harboring the recombineering system for homologous recombination (18). However, *in vivo* homologous recombination methods are still limited by the low editing frequency and the time-consuming screening process caused by the short lytic lifecycle of virulent phages in infected bacterial cells and the low efficiency of homologous recombination in most bacteria. Furthermore, multiplex engineering of the phage genome cannot be achieved via *in vivo* homologous recombination.

Alternatively, the phage genome can be separately amplified by PCR into several DNA fragments with terminal overlapping sequences and then assembled *in vitro* (19, 20) or in yeast (21). Multiple targeted mutations can be introduced during assembly. The phage genome can also be recoded via *de novo* chemical synthesis (22, 23). The edited phage genomic DNA is then transformed into bacterial host strains to reboot. Recently, a cell-free transcription‒translation system was developed to reboot *in vitro* assemblies of bacteriophage genomes from PCR fragments (17, 24). However, a variety of random mutations introduced during PCR or *de novo* chemical synthesis are incorporated into the synthetic phage genome in addition to the targeted desired mutations (17, 23). Synthetic phage genomes obtained via these methods must be fully sequenced for further genotype‒phenotype relationship analyses.

In this study, we circumvented the toxicity of phage gene products to *E. coli* cloning host cells by splitting the toxic genomic segments of the virulent ΦX174 and T7 phages into smaller fragments that can be cloned and inserted into single-copy bacterial artificial chromosome (BAC) vectors, which are efficiently genetically manipulated via homologous recombination-based recombineering (25) at the plasmid level. Knowledge of toxic genes within the phage genome is needed to guide segment splitting. Ten segments (1.2–7.8 kb) split from the 39.9-kb T7 phage genome and cloned and inserted into the BAC vectors were then simultaneously modified via recombineering (26) to achieve multiplex genome engineering. The linear genomic segments with 40 bp terminal overlapping sequences were released from the BAC vectors and then assembled via the ExoCET method (27, 28), which combines exonuclease *in vitro* assembly with RecET recombination for efficient multipiece DNA assembly, to obtain the intact genome and reboot the synthetic phage in *E. coli*. We termed this splitting-modifying-assembling-rebooting phage engineering method SMART. Using the SMART method, a T7 chassis phage with 10% genome reduction was constructed. The insertion capacity and expression level of exogenous genes at different T7 phage genomic loci were evaluated. To expand the lysis spectrum of the T7 phage, the genes encoding the lysins from the *S. aureus* and *S. agalactiae* phages were separately inserted into two genomic loci in the T7 chassis phage. The synthetic T7 phage lysed *E. coli*, *S. aureus,* and *S. agalactiae* when they were cultured together.

## RESULTS

### Splitting the virulent phage genome into segments clonable into *E. coli* vectors

**Fig 1:**
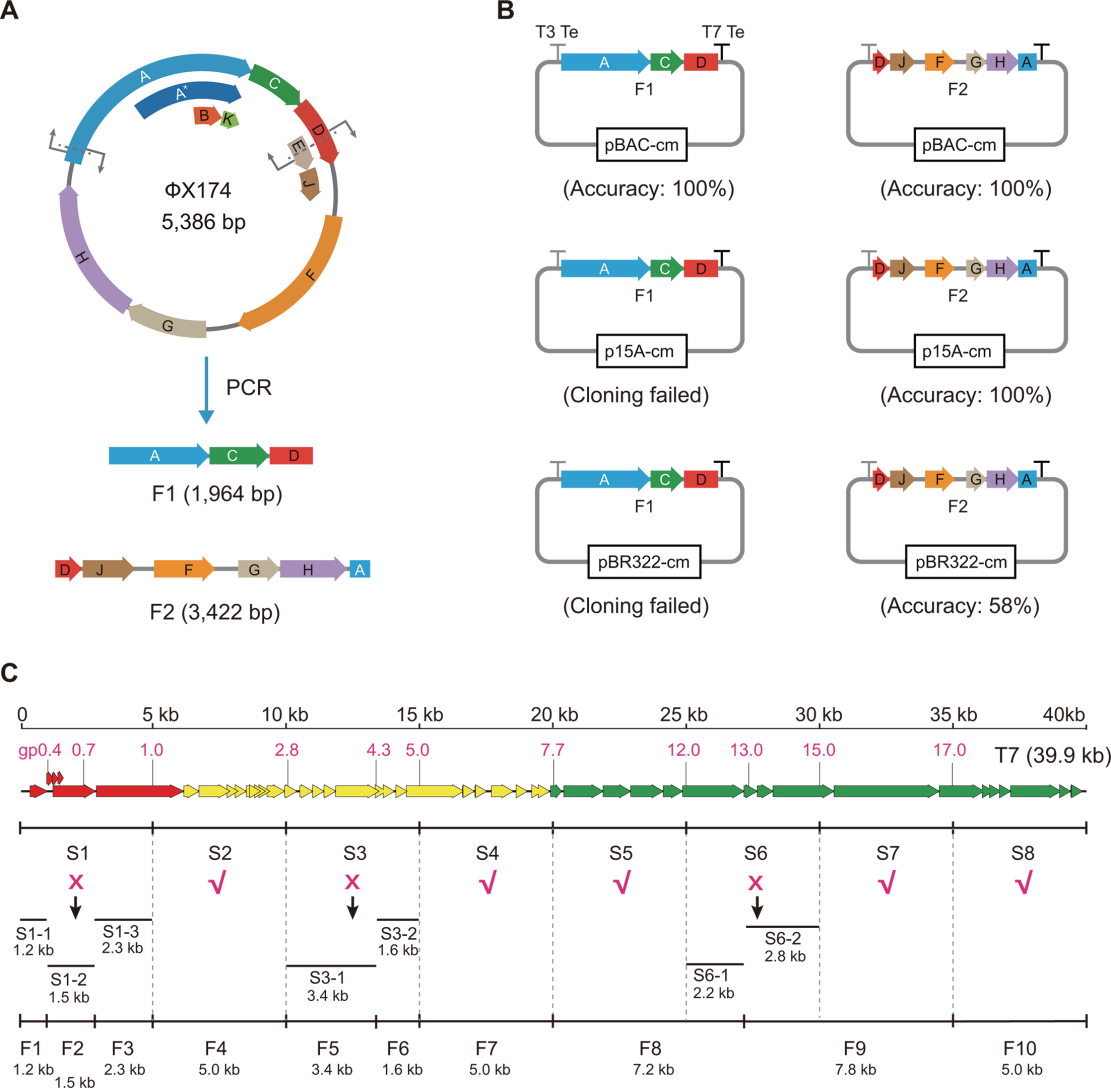
Splitting phage genomes into clonable segments. (**A**) Splitting the ΦX174 genome. (**B**) Efficiency of cloning the F1 and F2 segments into *E. coli* vectors with different copy numbers. T3Te, T3 terminator; T7Te, T7 terminator. (**C**) Splitting the T7 genome. The early, middle, and late genes in the T7 genome are shown in red, yellow, and green, respectively.

We first tried to clone the whole 5,386 bp genome of the ΦX174 phage (Fig. 1A) into the BAC vector by splitting gene E, which encodes the cell lysis protein. The linear BAC vector was inserted between the 92nd and 93rd nucleotides of gene E (201 bp) to disrupt its open reading frame. However, no correct clones were obtained; while a few colonies were obtained on the selection plate, all of them contained the empty vector (Fig. S1). This result indicated that other toxic components in addition to the cell lysis protein E encoded by the ΦX174 genome are lethal to *E. coli*. Splitting the ΦX174 genome into smaller segments and cloning these segments in separate vectors to replicate in individual cells may disrupt the production of toxic components. To do so, we split the 5,386 bp ΦX174 genome into a 1,964 bp segment, containing a partial gene A, the genes B, K, and C, a partial gene D, and a partial gene E, and a 3,422 bp segment, containing a partial gene D, a partial gene E, the genes J, F, G, and H, and a partial gene A (Fig. 1A). A terminator was placed at each end of the BAC vector to insulate the ΦX174 fragments from the neighboring promoters on the BAC vector. Both the PCR-amplified 1,964 bp (F1) and 3,422 bp (F2) segments were successfully cloned and inserted into the BAC vector with 100% accuracy (Fig. 1B; Fig. S2). The sequence correctness of the 1,964 bp and 3,422 bp ΦX174 genomic fragments cloned and inserted into the BAC vector was confirmed via Sanger sequencing.

Cloning of DNA fragments into higher-copy-number vectors will facilitate their preparation. We then tried to clone these two ΦX174 fragments into *E. coli* vectors with relatively high copy numbers. The low-copy p15A vector (10–12 copies per cell) (29) and the medium-copy pBR322 vector (15–20 copies per cell) (29) were used. A terminator was also placed at each end of the p15A or pBR322 vector to insulate the ΦX174 fragments from the neighboring promoters on the vectors. The 3,422-bp F2 segment was cloned and inserted into the p15A vector with 100% accuracy, whereas it was cloned and inserted into the pBR322 vector with significantly reduced (58%) accuracy (Fig. 1B; Fig. S2). Cloning of the 1,964-bp F1 segment into either the p15A vector or the pBR322 vector failed (Fig. 1B; Fig. S2). pBAC-ΦX174-F1 and p15A-ΦX174-F2 have been deposited at Addgene. Their nucleic acid sequences are also available in the database of the National Center for Biotechnology Information (NCBI). The Addgene IDs and NCBI accession numbers are provided in Table S1. The above results suggested that the genomes of virulent *E. coli* phages can be cloned and inserted into *E. coli* vectors by splitting them into smaller clonable segments. Cloning phage genomic segments into a single-copy BAC vector can minimize the production of toxic components and prevent lethal effects on *E. coli* cloning hosts.

We then tested the feasibility of cloning larger phage genomes. The 39.9-kb T7 phage genome was first split into eight ~5 kb segments (S1–S8, Fig. 1C), each of which was amplified via PCR and then cloned and inserted into the BAC vector via RecET-mediated linear‒linear homologous recombination in *E. coli* GB05-dir. The results revealed that the S2, S4, S5, S7, and S8 segments were successfully cloned and inserted into the BAC vector and that the BAC vector containing these fragments stably replicated in *E. coli* GB05-dir (Fig. 1C; Fig. S3). However, the cloning of the S1, S3, and S6 segments failed.

The S1, S3, and S6 segments were then further split into smaller segments. The *gp0.7* gene in S1, encoding a protein kinase that interacts with the host, is potentially toxic to *E. coli* GB05-dir. S1 was split into two segments by destroying the *gp0.7* gene; however, cloning of these two smaller segments still failed. According to previously published results, the 1.5 kb region in the middle of S1 is dispensable for T7 phage; therefore, we separately amplified the 1.2 kb left S1-1 region, the 1.5 kb middle S1-2 region, and the 2.3 kb right S1-3 region via PCR and found that these three smaller segments could be cloned and inserted into the BAC vector (Fig. 1C; Fig. S4). Neither S1-1 and S1-2 nor S1-2 and S1-3 could be fused to the clone in a single BAC vector. Additionally, S1-3 cannot be further fused with S2.

The S3 segment was split into two 2.5 kb segments, and we found that the left segment could be cloned and inserted into the BAC vector, whereas the right segment could not be. The right 2.5-kb S3 fragment was then further split into 0.9 kb and 1.6 kb segments, both of which were then successfully cloned and inserted into the BAC vector. The remaining 2.5 kb segment was subsequently fused with the adjacent 0.9 kb segment to obtain the 3.4 kb S3-1 segment, which could be cloned and inserted into the BAC vector (Fig. 1C; Fig. S5). The 3.4-kb S3-1 segment and the 1.6-kb S3-2 segment could not be further fused with the adjacent S2 or S4 segments.

The S6 segment consists of structural genes, and there are no known toxic genes in this segment. We split S6 into a 2.2 kb segment (S6-1) and a 2.8 kb (S6-2) segment by destroying the *gp13* gene, which encodes the internal virion protein A. Both S6-1 and S6-2 were successfully cloned and inserted into the BAC vector (Fig. 1C; Fig. S6). S6-1 and S6-2 were then successfully fused with the adjacent 5 kb S5 and S7 segments to obtain 7.2 kb and 7.8 kb fragments, respectively, which could be cloned and inserted into the BAC vector (Fig. 1C; Fig. S7). Finally, we split the 39.9-kb T7 phage genome into 10 1.2 kb–7.8 kb clonable segments (F1–F10), which could not be further fused with adjacent segments. BAC constructs containing these 10 clonable T7-phage segments have been deposited at Addgene. Their nucleic acid sequences are also available in the NCBI database. The Addgene IDs and NCBI accession numbers are provided in Table S1.

### Rebooting phage virions by assembling split segments

Each of the ΦX174 or T7 phage genomic segments cloned and inserted into the BAC vectors was flanked with BstZ17I or NaeI restriction sites so that they could be released from the BACs. Furthermore, each phage genomic segment carries 40 bp overlaps between adjacent fragments so that they can be assembled into intact phage genomes via homologous recombination-based assembly methods. In this study, we compared three assembly methods to reboot phage virions. (i) *In vitro* homologous recombination is mediated by Gibson assembly (30). The mixture of phage genomic segments was treated using the Gibson assembly kit and then electroporated into *E. coli* GB2005 (25). (ii) *In vivo* homologous recombination is mediated by RecET in *E. coli* (31). The mixture of phage genomic segments was electroporated into *E. coli* GB05-dir (31) expressing the RecET recombinases. (iii) ExoCET (*in vitro + in vivo*) homologous recombination is achieved by combining Gibson assembly and RecET assembly in *E. coli* (28). The mixture of phage genomic segments was treated using the Gibson assembly kit and then electroporated into *E. coli* GB05-dir + pSC101-BAD-ETgA-tet (28) expressing RecET recombinases.

**Fig 2:**
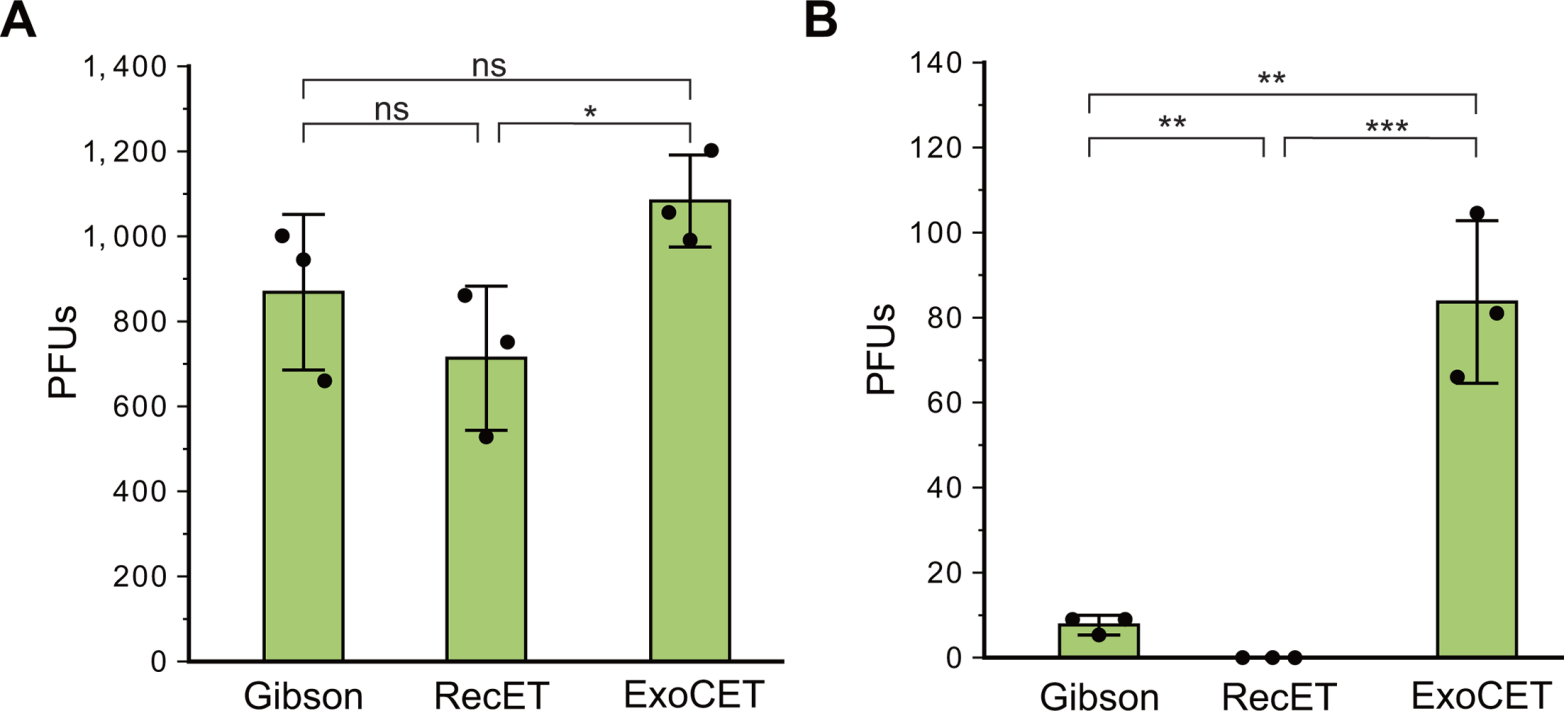
Efficiency of rebooting phage virions by assembling split genomic segments via three methods. (**A**) Plaque-forming units (PFUs) of ΦX174 rebooted by assembling the F1 (100 ng) and F2 (100 ng) segments. (**B**) PFUs of T7 rebooted by assembling the F1‒F10 segments (F1 100 ng, F2 100 ng, F3 100 ng, F4 150 ng, F5 100 ng, F6 100 ng, F7 150 ng, F8 150 ng, F9 150 ng, F10 150 ng). The CFUs of *E. coli* used in the transformations were normalized to 8 × 10^8^ CFUs mL^−1^ across methods. *n* = 3 independent experiments. The data are presented as the means ± S.D.s. Statistical analysis comparing two groups was performed using a two-sided Student’s *t* test. *P < 0.05; **P < 0.01; ***P < 0.001; ns, not significant.

When the ΦX174 phage was rebooted by assembling the two segments, the *in vitro + in vivo* ExoCET method was the most efficient. A total of 868 ± 182 plaque-forming units (PFUs) were obtained via the *in vitro* Gibson method, 713 ± 169 PFUs were obtained via the *in vivo* RecET method, and 1,083 ± 108 PFUs were obtained via the *in vitro + in vivo* ExoCET method (Fig. 2A). When the T7 phage was rebooted by assembling the 10 segments (F1–F10), the *in vitro + in vivo* ExoCET method was also the most efficient. A total of 8 ± 2 PFUs were obtained via the *in vitro* Gibson method, whereas the *in vivo* RecET method failed, and 84 ± 19 PFUs were obtained via the *in vitro + in vivo* ExoCET method (Fig. 2A). Therefore, when the phage genome is split into clonable segments, the phage virion can be rebooted by assembling these segments via the *in vitro + in vivo* ExoCET method with high efficiency.

### T7 phage genome reduction by simultaneous engineering of genomic segments cloned in the BAC vector

**Fig 3:**
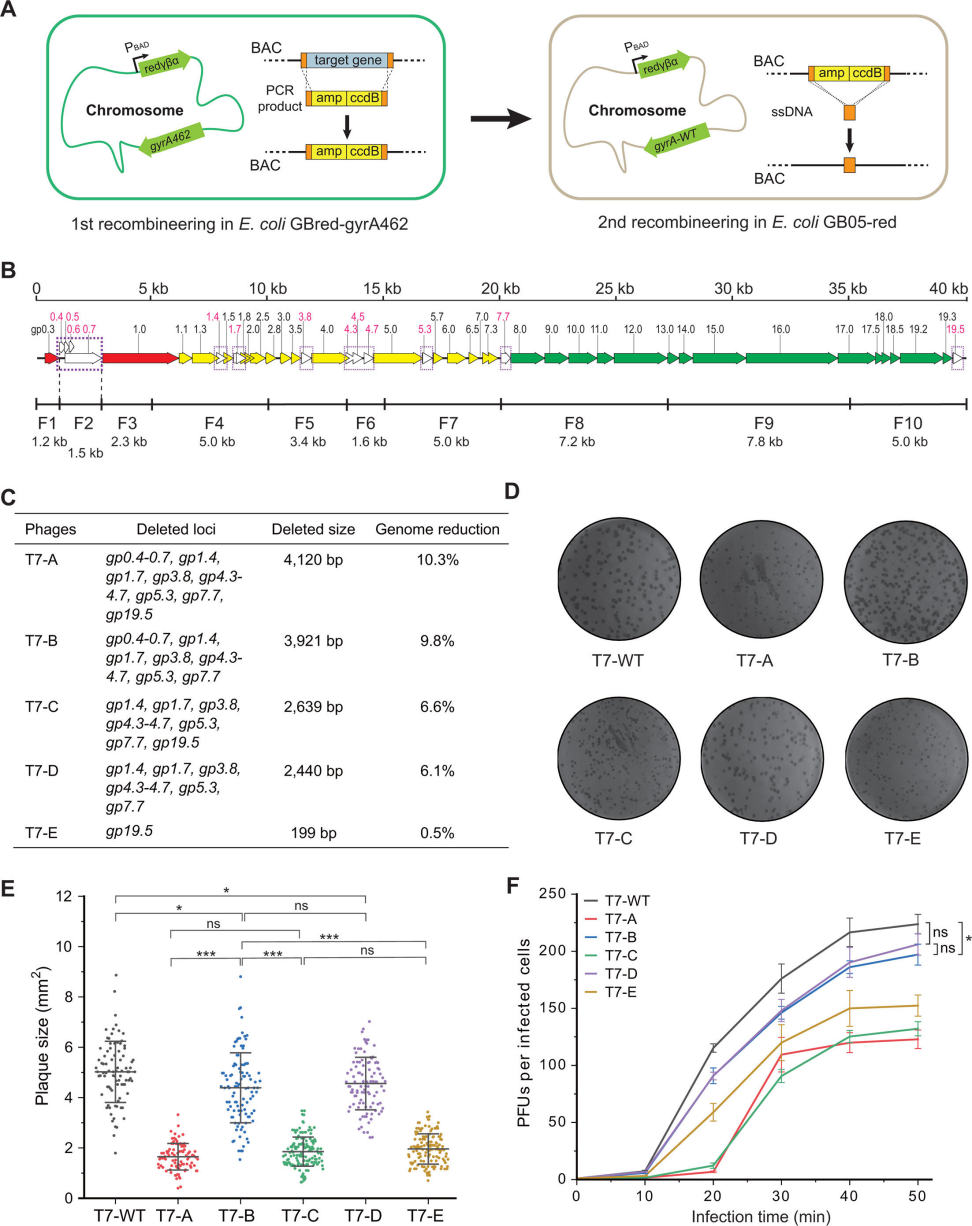
phage genome reduction. (**A**) Overview of the Red-ccdB method for seamless site-directed mutagenesis. (**B**) Schematic diagram of the T7 genome. Nonessential genes are shown as open arrows, and deleted regions are marked with dotted boxes. (**C**) T7 phage mutants constructed in this study. (**D**) Plaques of T7 wild-type and mutated phages. (**E**) Scatter plot graphs showing plaque areas of T7 wild-type and mutated phages. Plaque areas were measured using ImageJ software. The data are presented as the means ± S.D.s. The horizontal bars indicate the means of the data set. The vertical bars indicate the error bars (S.D.s). (**F**) One-step growth curves of T7 wild-type and mutated phages. *n* = 3 independent experiments. The data are presented as the means ± S.D.s. Statistical analyses were performed using OriginPro 2021b software. Statistical analysis comparing two groups was performed using a two-sided Student’s *t* test. *P < 0.05; ***P < 0.001; ns, not significant.

Nonessential genes of the T7 phage were previously systematically identified (Table S2), and T7 mutants with reduced genomes were generated by deleting several nonessential genes (32). However, the T7 minimal genome without all nonessential genes has not yet been constructed. All genes (*gp0.4~gp*.7) in the F2 fragment were identified as nonessential (32). The *gp1.4* gene and the *gp1.7* gene on the F4 segment, the *gp3.8* gene on the F5 segment, the *gp4.3‒gp4.7* genes on the F6 segment, the *gp5.3* gene on the F7 segment, the *gp7.7* gene on the F8 segment, and the *gp19.5* gene on the F10 segment were deleted seamlessly via the Red-ccdB selection–counterselection-based two-round recombineering method (25) (Fig. 3A and B). In the first round of recombineering, a cassette consisting of the ampicillin resistance gene (*amp*, the selection marker) and the *ccdB* toxin gene (the counterselection marker) was inserted at the target site in the T7 phage genomic segment on the BAC vector in *E. coli* GBred-gyrA462. The GyrA subunit of DNA gyrase is the target of CcdB toxin, and *E. coli* GBred-gyrA462 carries the gyrA_Arg462Cys_ mutation, which confers CcdB resistance. In the second round of recombineering, synthetic single-stranded oligonucleotides consisting of two 40-nt homology arms were used to replace the *amp-ccdB* cassette in *E. coli* GB05-red, which is sensitive to the CcdB toxin because its GyrA subunit is wild type (Fig. 3A).

Unmodified F1, F3, and F9 segments were mixed with modified F4‒F8 segments, and the F10 segments containing gene deletions were assembled and rebooted via the ExoCET method. A viable phage (T7-A) with a 35.8 kb genome (10.3% reduction) was thus created (Fig. 3C). However, the plaque size of T7-A was much smaller than that of wild-type T7 (Fig. 3D and E). To determine which gene deletion reduced the infectious efficiency, we changed the combination of T7 cloned genomic segments on the basis of the T7-A assembly as follows: (i) Unmodified F10 was used to replace the modified F10 to create the phage mutant T7-B. (ii) Unmodified F2 was added to create the phage mutant T7-C. (iii) Unmodified F2 was added, and unmodified F10 was used to replace the modified F10 to create the phage mutant T7-D (Fig. 3C). The results revealed that the plaque sizes of T7-B and T7-D were comparable to that of wild-type T7, whereas the plaque size of T7-C was comparable to that of T7-A, which was much smaller than that of wild-type T7 (Fig. 3D and E). We then mixed unmodified F1–F9 and modified F10 to assemble and reboot via the ExoCET method. A viable phage (T7-E) with the *gp19.5* gene deletion was obtained (Fig. 3C). The plaque size of T7-E was comparable to that of T7-A and T7-C, which were much smaller than that of wild-type T7 (Fig. 3D and E). The above deletions in the T7 phage genome were confirmed via PCR (Fig. S8) and whole-genome sequencing. Therefore, the deletion of the *gp19.5* gene only significantly reduces plaque size. The *gp19.5* gene is a hypothetical gene, and its function is unknown (Table S3).

One-step growth experiments suggested that the burst sizes of T7 mutants with reduced genomes all decreased compared with those of the T7 wild-type phage (224 ± 9). The burst size of T7-A decreased to 122 ± 9, that of T7-B decreased to 197 ± 9, that of T7-C decreased to 132 ± 6, that of T7-D decreased to 206 ± 10, and that of T7-E decreased to 152 ± 9 (Fig. 3F). Finally, we chose the T7-B mutant with the 36.0 kb genome (9.8% reduction) as the chassis phage while considering the trade-off between phage genome reduction and infectious efficiency. By releasing 3.9 kb from the 39.9 kb wild-type T7 genome, the T7-B chassis phage could be engineered to express large exogenous gene inserts. The genome sequence of the T7-B chassis phage has been uploaded to NCBI, and its accession number is provided in Table S1.

### Comparison of exogenous gene expression levels at different loci in the T7-B chassis phage genome

**Fig 4:**
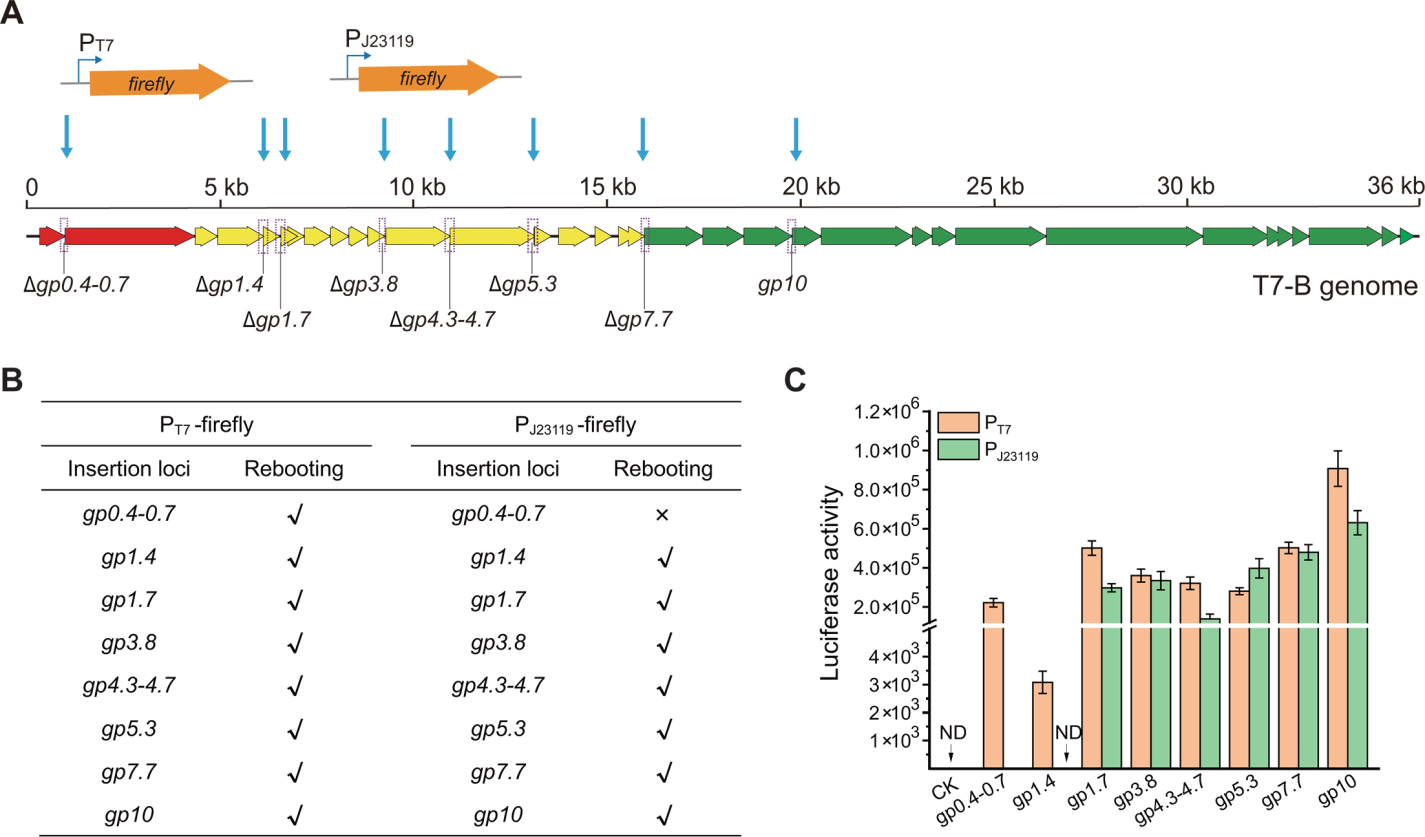
Expression of the *firefly* luciferase gene at different loci in the T7-B genome. (**A**) Insertion of the *firefly* luciferase gene under the control of the T7 or J23119 promoter at different loci in the T7-B genome. (**B**) Effects of *firefly* luciferase gene insertion on phage rebooting. (**C**) Luciferase activity of T7-B-mutated phages with the *firefly* luciferase gene inserted at different loci. The T7-B phage was used as a control (CK) for the luciferase activity assay. ND, no luciferase activity was detected. *n* = 3 independent experiments. The data are presented as the means ± S.D.s.

In chassis phages, the genomic loci used to insert exogenous genes for expression should be characterized. We analyzed the expression of the *firefly* luciferase reporter gene at the seven deleted loci in the T7-B genome (Fig. 4A). In the T7 phage display system, target peptides or proteins are displayed on the surface of the T7 phage via carboxyl-terminal fusion to the capsid protein Gp10B (33). Furthermore, the locus between the *gp10* and *gp11* genes in the T7 phage genome is usually used to integrate reporter genes to construct recombinant phages for *E. coli* detection (34, 35). Therefore, the expression of the *firefly* luciferase reporter gene at the *gp10* locus was also analyzed (Fig. 4A). The *firefly* luciferase reporter gene under the control of the T7 phage promoter or the J23119 promoter was inserted seamlessly at each locus in the cloned segments via the Red-ccdB method. The T7 phage promoter is recognized by T7 phage RNA polymerase but not by bacterial RNA polymerases (36). The T7 RNA polymerase/T7 promoter system is commonly used for controlling the exclusive expression of genes cloned downstream of the T7 promoter. The J23119 promoter is a synthetic strong constitutive *E. coli* promoter (37, 38) that is recognized by the *E. coli* endogenous RNA polymerase. In the second round of recombineering, the P_T7_-firefly or P_J23119_-firefly cassettes flanked by 40 bp homology arms were used to replace the *amp-ccdB* cassette at each locus in the cloned segments in *E. coli* GB05-red.

After genomic segment assembly and phage rebooting, viable recombinant phages carrying the P_T7_-firefly cassette at each of the eight loci were obtained (Fig. 4B). Insertion of the P_J23119_-firefly cassette at the *gp0.4-gp0.7* locus failed to yield a viable phage; however, insertion of the P_J23119_-firefly cassette at the other seven loci resulted in viable phages (Fig. 4B). The luciferase activity of P_T7_-firefly at the *gp1.4* locus was the lowest, and that at the *gp10* locus was the highest. The luciferase activity of P_T7_-firefly at the *gp10* locus was 294.4 times greater than that at the *gp1.4* locus. The luciferase activity of P_J23119_-firefly at the *gp1.4* locus was undetectable, whereas that at the *gp10* locus was highest. The luciferase activity of the P_J23119_-firefly at the *gp10* locus was 4.6 times greater than that at the *gp4.3-gp4.7* locus. At the *gp1.7*, *gp4.3-gp4.7,* and *gp10* loci, the luciferase activities of P_T7_-firefly were greater than those of P_J23119_-firefly. At the *gp3.8* and *gp7.7* loci, the luciferase activities of P_T7_-firefly were identical to those of P_J23119_-firefly. At the *gp5.3* locus, the luciferase activity of P_T7_-firefly was lower than that of P_J23119_-firefly (Fig. 4C).

### Evaluation of the exogenous gene insertion capacity at different loci in the T7-B chassis phage genome

**Fig 5:**
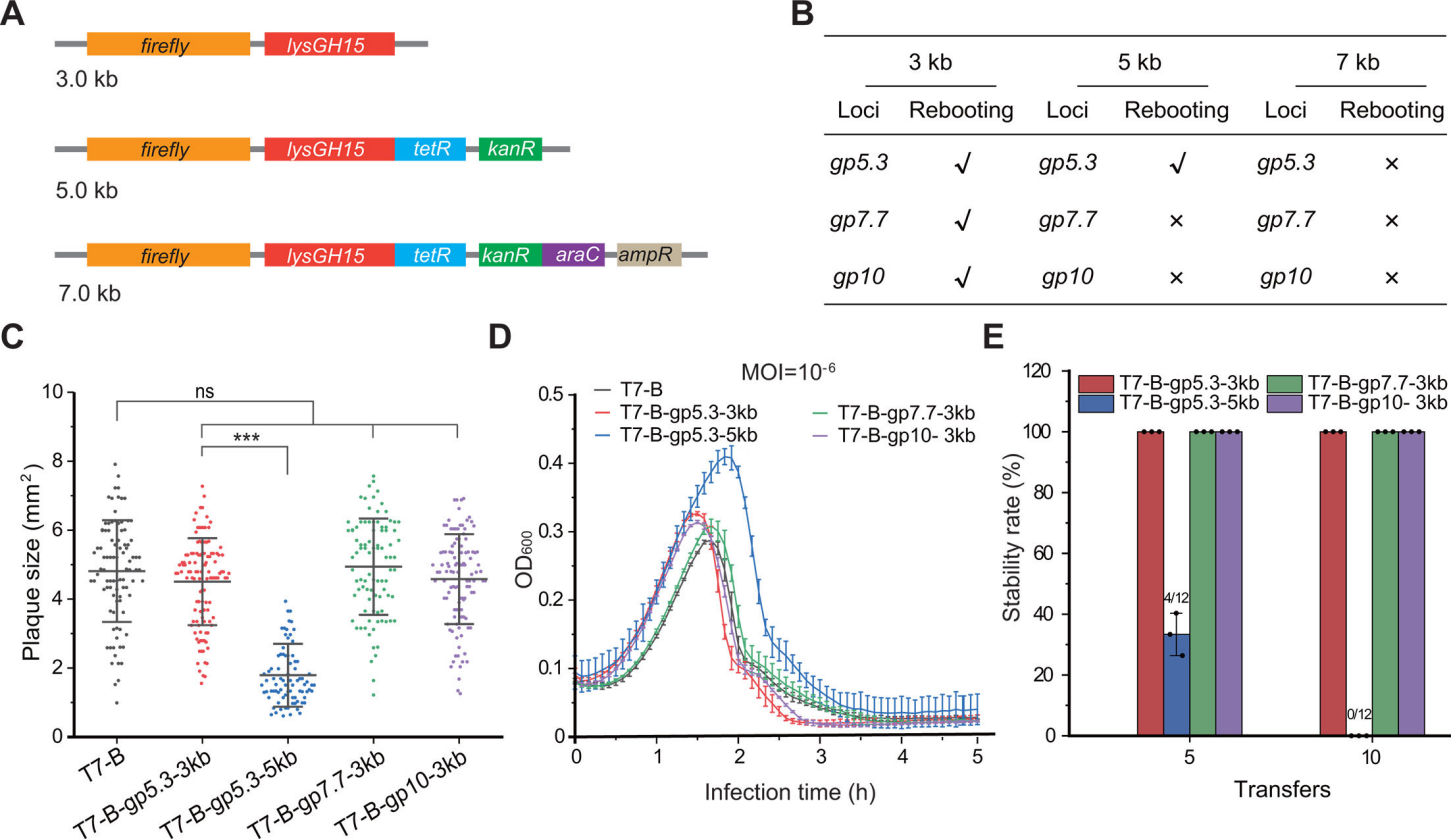
Evaluation of the exogenous gene insertion capacity of the T7-B chassis phage genome. (**A**) Schematic diagram of the 3 kb, 5 kb, and 7 kb exogenous gene inserts used in this study. (**B**) Effects of exogenous gene inserts on phage rebooting. (**C**) Scatter plot graphs showing plaque areas of T7-B variants. Plaque areas were measured using ImageJ software. The data are presented as the means ± S.D.s. The horizontal bars indicate the means of the data set. The vertical bars indicate the error bars (S.D.s). Statistical analyses were performed using OriginPro 2021b software. Statistical analysis comparing two groups was performed using a two-sided Student’s *t* test. ***P < 0.001; ns, not significant. (**D**) Phage infection growth curves of the T7-B variants with exogenous gene inserts. *n* = 3 independent experiments. The data are presented as the means ± S.D.s. (**E**) Genetic stability of T7-B-mutated phages with exogenous gene inserts. Twelve phages of each T7-B mutant were randomly selected, and the 3 kb and 5 kb exogenous DNA fragments were checked via PCR (Fig. S9). The stability rate is defined as the number of phages containing foreign DNA fragments out of 12 phages. *n* = 3 independent experiments. The data are presented as the means ± S.D.s.

We selected the *gp5.3*, *gp7.7*, and *gp10* loci, which exhibited relatively high expression levels of the *firefly* luciferase reporter gene, to evaluate the exogenous gene insertion capacity. DNA fragments (3 kb, 5 kb, and 7 kb) with similar GC contents (~45%) to those of the T7 phage genome (47%) were prepared (Fig. 5A) via overlap extension PCR. The 3 kb fragment consists of a partial *firefly* luciferase gene and a partial *lysGH15* lysin gene. The 5 kb fragment consists of the 3 kb fragment, a partial tetracycline resistance gene, and a partial kanamycin resistance gene. The 7 kb fragment consists of the 5 kb fragment, a partial *araC* gene, and a partial ampicillin resistance gene. All the open reading frames on the above fragments were destroyed to prevent their products from affecting phage viability. The 3 kb, 5 kb, and 7 kb DNA fragments were successfully inserted seamlessly at the *gp5.3*, *gp7.7*, and *gp10* loci, respectively, into the corresponding cloned genomic segments in the BAC vector via the Red-ccdB method. After genomic segment assembly and phage rebooting, no viable recombinant phages were obtained in the assembly combination for the wild-type T7 phage. Therefore, inserts larger than 3 kb cannot be inserted into the wild-type T7 phage genome.

Viable recombinant phages carrying 3 kb DNA inserts at all three tested loci were obtained in the assembly combination for the T7-B phage genome. The 7 kb DNA fragments could not be inserted into three tested loci in the T7-B genome. The 5 kb DNA inserts were successfully inserted into the *gp5.3* locus but could not be inserted into the *gp7.7* and *gp10* loci in the T7-B genome (Fig. 5B). The infection efficiency of T7-B-gp5.3-5 kb is much lower than those of T7-B-gp5.3-3 kb, T7-B-gp7.7-3 kb, and T7-B-gp10-3 kb, which are similar to those of T7-B (Fig. 5C and D).

The genetic stability of the recombinant phages T7-B-gp5.3-3 kb, T7-B-gp5.3-5kb, T7-B-gp7.7-3kb, and T7-B-gp10-3kb was evaluated by performing 10 transfers (60–80 generations) of them into the host *E. coli* BL21(DE3). The inserts on the phage genomes were detected via PCR. The results suggested that the 3 kb insert was stably maintained at the *gp5.3*, *gp7.7*, and *gp10* loci after 10 transfers. However, the 5 kb insert at the *gp5.3* locus could not be detected in 67% (8/12) of the randomly chosen phages after five transfers. After 10 transfers, the 5 kb insert at the *gp5.3* locus could not be detected in any of the 12 randomly chosen phages (Fig. 5E; Fig. S9). Therefore, the T7-B chassis phage with 9.8% genome reduction can stably carry 3 kb inserts at the *gp5.3*, *gp7.7*, and *gp10* loci.

### Construction of synthetic T7 phages that lyse bacteria from different phyla

After determining the exogenous gene expression level and insertion capacity at different loci in the T7-B chassis phage genome, we assessed its ability as a scaffold to carry different payload genes to target polymicrobial communities. As a model system, we constructed synthetic T7 phages expressing lysins that recognize and degrade the cell walls of the pathogenic bacteria *S. aureus* (phylum Bacillota) or *S. agalactiae* (phylum Bacillota). Lysins can be expressed during infection by synthetic T7 phages in *E. coli* (phylum Pseudomonadota) and are released upon lysis of *E. coli*. The released lysins then lyse bystander *S. aureus* or *S. agalactiae* cells. To achieve this lysis, the LysGH15 lysin against *S. aureus* and the LyJDSS3 lysin against *S. agalactiae* were used (39).

**Fig 6:**
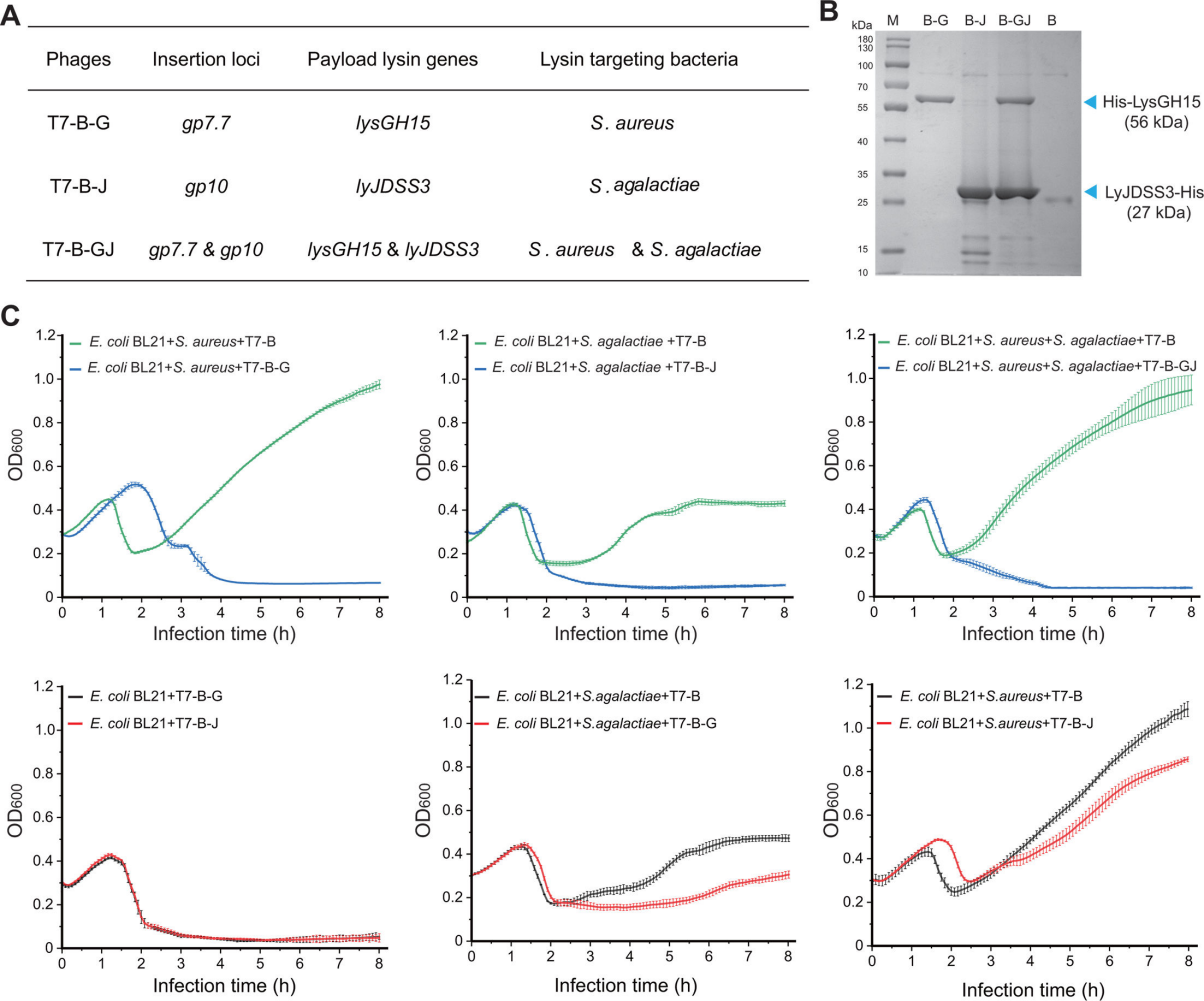
Evaluation of the expression and activity of heterologous lysins from synthetic T7 phages. (**A**) T7-B-mutated phages constructed in this study to express heterologous lysins. (**B**) SDS-PAGE analysis of LysGH15 and LyJDSS3 proteins expressed from *E. coli* BL21(DE3) infected with T7-B-G, T7-B-J, and T7-B-GJ synthetic phages. (**C**) Lysate curve of bacterial cells in coculture with synthetic phages expressing heterologous lysins. *n* = 3 independent experiments. The data are presented as the means ± S.D.s.

Because the expression levels of the *firefly* luciferase gene at the *gp7.7* and *gp10* loci in the T7-B chassis genome were highest (Fig. 4C), we inserted the lysin genes under the control of the T7 promoter at these two loci. The 1.6 kb P_T7_-*lysGH15* cassette was inserted at the *gp7.7* locus to obtain the synthetic phage T7-B-G, and the 0.8 kb P_T7_-*lyJDSS3* cassette was inserted at the *gp10* locus to obtain the synthetic phage T7-B-J (Fig. S10). The synthetic phage T7-B-GJ was constructed to carry both the P_T7_-*lysGH15* cassette at the *gp7.7* locus and the P_T7_-*lyJDSS3* cassette at the *gp10* locus (Fig. 6A). A 6× His tag was fused to the N-terminus of LysGH15 and the C-terminus of LyJDSS3 to facilitate their purification and detection of their production during infection via SDS‒PAGE. The genome sequences of the synthetic T7 phages T7-B-G, T7-B-J, and T7-B-GJ have been uploaded to NCBI, and their accession numbers are provided in Table S1.

The His-tagged lysins were purified via affinity chromatography from the lysate of *E. coli* BL21(DE3) infected with the T7-B-G, T7-B-J, or T7-B-GJ phages. The SDS-PAGE results revealed bands corresponding to the predicted molecular weight of the His-LysGH15 protein in the lysate of T7-B-G, the LyJDSS3-His protein in the lysate of T7-B-J, and both the His-LysGH15 protein and LyJDSS3-His protein in the lysate of T7-B-GJ. These bands were all absent from the T7-B lysate (Fig. 6B).

For the coculture infection experiments, mixtures of *E. coli* BL21(DE3) and *S. aureus* ATCC 25923 or *S. agalactiae* ATCC13813 were inoculated with the synthetic T7 phages at an MOI of 0.001. An 8 h turbidity reduction assay revealed that T7-B-G can infect and kill *E. coli* cells while subsequently killing cocultured *S. aureus* through *in situ* release of the LysGH15 lysin. T7-B-J can infect and kill *E. coli* cells while subsequently killing cocultured *S. agalactiae* through *in situ* release of LyJDSS3. T7-B-GJ can infect and kill *E. coli* cells while subsequently killing cocultured *S. aureus* and *S. agalactiae* through *in situ* release of LysGH15 and LyJDSS3 (Fig. 6C). When T7-B-G expressing the LysGH15 lysin against *S. aureus* was added to a mixture of *E. coli* BL21(DE3) and *S. agalactiae* ATCC13813, the growth of *S. agalactiae* ATCC13813 was inhibited. When T7-B-J expressing the LyJDSS3 lysin against *S. agalactiae* was added to a mixture of *E. coli* BL21(DE3) and *S. aureus* ATCC 25923, the growth of *S. aureus* ATCC 25923 was also inhibited. The results suggested that neither of the above lysins is exclusively specific to each bacterium.

The genetic stability of T7-B-G, T7-B-J, and T7-B-GJ was determined by transferring them 10 times (60–80 generations) into the host *E. coli* BL21(DE3). The turbidity reduction assay revealed that 10 out of the 10 checked T7-B-G, T7-B-J, and T7-B-GJ progeny phages maintained their lysis activity toward the corresponding bacteria after 10 transfers (Fig. S11). PCR amplification and sequencing of the lysin genes in the genomes of the T7-B-G, T7-B-J, and T7-B-GJ progeny phages also confirmed that the lysin genes were stably maintained at the corresponding loci (Fig. S12).

## DISCUSSION

Synthetic biology approaches could revolutionize phage therapy by allowing for the design and engineering of phages with improved lysis properties (40, 41). This requires a thorough understanding of the phage genome structure and function, as well as the interaction mechanisms between phages and both bacterial and animal hosts. To achieve this goal, rapid, precise, and efficient genome engineering technologies are essential (42).

The major advantage of SMART over existing approaches is that the entire virulent phage genome can be split into segments that can be cloned in BACs. To our knowledge, no previous studies have successfully accomplished this goal because virulent phage genes usually encode products that are toxic to the *E. coli* cloning host. The 30 kb SARS-CoV-2 cDNA genome has been split across seven plasmids, engineered and rebooted to establish a reverse genetic system due to the presence of several toxic genomic elements (43, 44). This research inspired us to split the virulent phage genome across BACs. The advantages of splitting the genome into BACs include the following. (i) Compared with the preparation of genomic fragments via PCR or *de novo* chemical synthesis, the virulent phage genomic segments cloned in BACs can be stably propagated in *E. coli* cells with high fidelity; therefore, the engineered phages do not need to be fully sequenced for further genotype‒phenotype relationship analysis. Only the engineered loci on the respective BAC need to be sequenced. (ii) Compared with the BRED method, SMART allows multiplex engineering, and its screening process is very simple because it reboots the engineered phage by transforming the assembly of genomic segments that are already genetically modified. No wild-type phage was included in the screening.

Disruption of the production of the toxic components encoded by the DNA regions in the virulent phage genome is critical to successfully establish the SMART method for a specific virulent phage. However, most of the toxic components encoded by the phage genome have not been characterized, and how they are produced and how their production is regulated are unclear. Currently, we cannot predict which genomic segments should be split and which two adjacent genomic segments can be fused, but we have found that early and middle genes involved in the interaction with the host have a relatively high probability of being toxic genes and require more segmentation to eliminate their toxicity. Although the splitting strategy for a particular phage genome must be determined case by case, the SMART method enriches the toolbox of phage genome editing.

Previous studies have shown that engineered phages carrying genes encoding foreign effectors, including depolymerases (6), antimicrobial peptides (45), bacteriocins (41), and the CRISPR‒Cas system (46), have greater bactericidal ability and broader spectral activity. However, the capacity of the phage genome to carry foreign genes is generally limited. In this study, the foreign 3 kb DNA fragment could not be inserted into the wild-type T7 phage genome at the *gp5.3*, *gp7.7*, and *gp10* loci. However, the T7-B chassis phage with 9.8% genome reduction can stably carry 3 kb inserts at these three loci (Fig. 5). Therefore, it is particularly important to reduce the size of the phage genome and develop chassis phages that express foreign genes efficiently. The high efficiency and high fidelity of the SMART method can compensate for the shortcomings of existing tools.

## MATERIALS AND METHODS

### Bacteria culture conditions

*E. coli* strains (Table S4) were cultured at 30°C or 37°C in LB medium supplemented with the appropriate antibiotics (15 µg mL^−1^ chloramphenicol, 100 µg mL^−1^ ampicillin, or 5 µg mL^−1^ tetracycline). *S. aureus* ATCC 25923 was cultured in LB medium at 37°C. *S. agalactiae* ATCC13813 was cultured in brain heart infusion (BHI) medium at 37°C.

### Preparation of phage genomic DNA

Fifty milliliters of log-phase *E. coli* cells was infected with ΦX174 or T7 phages at a multiplicity of infection (MOI) of 0.01–0.001 and incubated until the culture became clear. The lysate was centrifuged at 8,300 × *g* for 5 min, and the supernatant was filtered through a 0.22 µm membrane to remove cell debris. Five microliters of 20 mg mL^−1^ RNaseA and 1 µL of 1 mg mL^−1^ DNase I were added to the filtrate and incubated at room temperature for 30 min. Then, 2.92 g NaCl was added, and the mixture was dissolved in the solution, which was further incubated at 4°C for 1 h. Five grams of polyethylene glycol 8000 was dissolved in the solution, which was incubated overnight at 4°C. The mixture was subsequently centrifuged at 9,500 × *g* for 30 min at 4°C to pellet the phage virions, which were then resuspended in 3 mL of ddH_2_O. Five hundred microliters of the phage virion suspension was transferred into a 1.5 mL tube, and 30 µL of 20 mg mL^−1^ proteinase K and 40 µL of 10% SDS were added. The mixture was incubated at 50°C for 30 min until the solution became clear. Six hundred microliters of the DNA extraction mixture (phenol, chloroform, and isoamyl alcohol, 25:24:1, pH 8.0) was added and mixed thoroughly via inversion. The suspension was subsequently centrifuged at 9,500 × *g* for 30 min at 4°C. The upper layer was transferred into a new 2 mL tube, and the genomic DNA was precipitated with ethanol. Finally, the phage genomic DNA was dissolved in 400 µL of ddH_2_O.

### Cloning of the phage genomic segments into vectors

The phage genomic segments were amplified via PCR using phage genomic DNA as the template, *Apex*HF HS DNA Polymerase (Accurate Biotechnology Co., Ltd., cat. no. AG12207) and the oligonucleotides listed in Table S5. The PCR products were purified from agarose gels after electrophoresis using the TIANgel Purification Kit (TIANGEN, cat. no. DP219) according to the manufacturer’s instructions. The DNA on the column was eluted with ddH_2_O. The phage genomic segment PCR products were subsequently cloned and inserted into the BAC, p15A, or pBR322 vectors via RecET-mediated linear‒linear homologous recombination in *E. coli* GB05-dir (31). Colonies containing the correct recombinant plasmids were selected on LB selection plates containing chloramphenicol and then identified via colony PCR. The sequences of the cloned phage genomic segments on the plasmids were confirmed via Sanger sequencing.

### Rebooting phage virions by assembling split segments

BACs carrying ΦX174 or T7 inserts were digested with BstZ17I or NaeI, respectively, to release the genomic segments, which were then purified from agarose gels after electrophoresis using the TIANgel Purification Kit (TIANGEN, cat. no. DP219) according to the manufacturer’s instructions. The DNA on the column was eluted with ddH_2_O.

When the Gibson assembly method (30) was used to recombine the phage genomic segments, 10 µL of the mixture containing approximately 100 ng of each phage genomic segment was mixed with 10 µL of Gibson Assembly Master Mix (NEB, cat. no. E2611) and incubated at 50°C for 2 h in a thermocycler. The reacted products were desalted on Millipore membrane filters (Merck-Millipore, cat. no. VSWP01300) via drop dialysis against ddH_2_O. The desalted reaction products were electroporated into *E. coli* GB2005 (25) cells.

When RecET-mediated linear‒linear homologous recombination (31) was used to recombine the phage genomic segments, 10 µL of the mixture containing approximately 100 ng of each phage genomic segment was electroporated into *E. coli* GB05-dir cells expressing the RecET recombinases.

When the ExoCET method (28) was used to recombine the phage genomic segments, the desalted Gibson assembly reaction products were electroporated into logarithmic *E. coli* GB05-dir cells (~8 × 10^8^ colony-forming units (CFUs) mL^−1^) harboring the pSC101-BAD-ETgA-tet plasmid and expressing the RecET recombinases.

The CFUs of *E. coli* used in the transformations were normalized to 8 × 10^8^ CFUs mL^−1^ across methods. After electroporation, 200 µL of LB medium was added to the cuvette to resuspend the cells. To reboot the ΦX174 phage, the cell suspension was transferred into 100 µL of the *E. coli* DH5α overnight culture. To reboot the T7 phage, the cell suspension was transferred into 100 µL of the *E. coli* GB05-dir overnight culture. The mixture was added to 8 mL of LB soft agar (0.7% agar, wt/vol) warmed at 52°C, poured onto an LB plate, and incubated for 8 h at 37°C to obtain phage plaques.

T7 mutant phages (T7-A, T7-B, T7-C, T7-D, and T7-E) whose genomes were reduced were checked by PCR analysis and next-generation sequencing. Other rebooted phages were checked via PCR analysis and Sanger sequencing.

### Seamless site-directed mutagenesis of cloned phage genomic segments via the Red-ccdB method

The *amp-ccdB* cassette was amplified via PCR with p15A-amp-ccdB (25) as the template, *Apex*HF HS DNA Polymerase (Accurate Biotechnology Co., Ltd., cat. no. AG12207) and the oligonucleotides listed in Table S6. The *amp-ccdB* PCR products carry 40 bp homology arms to the target sites at both termini. The PCR products were purified using the TIANquick Mini Purification Kit (TIANGEN, cat. no. DP203) according to the manufacturer’s instructions. The DNA on the column was eluted with ddH_2_O.

The mixture of BACs carrying the T7 phage genomic segment, and the corresponding *amp-ccdB* PCR products were electroporated into *E. coli* GBred-gyrA462 (25) expressing the Redαβ recombinases. Colonies containing correct recombined BACs were selected on LB selection plates containing chloramphenicol and ampicillin and then identified via colony PCR. The sequences of the *amp-ccdB* cassette and the flanking homology arms were confirmed via Sanger sequencing.

When the nonessential genes were deleted in the T7 phage genome, the mixture of the 80-nt oligonucleotides listed in Table S7 and BACs carrying the T7 phage genomic segments containing the *amp-ccdB* cassette at the target loci were electroporated into *E. coli* GB05-red (25) expressing the Redαβ recombinases. When the *firefly* luciferase reporter genes, the 3 kb/5 kb/7 kb DNA fragments or the lysin genes were inserted into the T7 phage genome, the mixture of the PCR products or the synthesized DNA fragments, and BACs carrying the T7 phage genomic segments containing the *amp-ccdB* cassette at the target loci were electroporated into *E. coli* GB05-red expressing the Redαβ recombinases. Colonies containing correct recombined BACs were selected on LB selection plates containing chloramphenicol and then identified via colony PCR. The sequences of the targeted mutations were confirmed via Sanger sequencing.

The *firefly* luciferase reporter genes were amplified via PCR with the oligonucleotides listed in Table S8. The 3 kb/5 kb/7 kb DNA fragments were prepared via overlap extension PCR using the oligonucleotides listed in Table S9. The *lysGH15* and *lyJDSS3* genes (39) were synthesized by GENEWIZ Biotechnology Co. Ltd. (Suzhou, China). The PCR products were purified from agarose gels after electrophoresis using the TIANgel Purification Kit (TIANGEN, cat. no. DP219) according to the manufacturer’s instructions. The DNA on the column was eluted with ddH_2_O.

### Measurement of phage infection growth curves

To compare the infection efficiency of T7 wild-type and mutant phages, the OD_600_ value of the overnight culture of *E. coli* BL21(DE3) was measured. An appropriate volume of the overnight culture of *E. coli* BL21(DE3) was diluted in 10 mL of LB medium to an OD_600_ value of 0.1. Two hundred microliters of the diluted culture was dispensed into each well of a 96-well plate. Ten microliters of phage lysate was added to each well at an MOI of 0.001. The 96-well plates were placed into a TECAN Infinite 200 microplate reader, and the OD_600_ was measured every 5 min at 37°C with agitation.

To evaluate the lysing activities of the T7 mutant phages expressing heterologous lysins, the OD_600_ values of the overnight cultures of *E. coli* BL21(DE3), *S. aureus* ATCC 25923, and *S. agalactiae* ATCC13813 were measured. An appropriate volume of the overnight culture of each strain was diluted in 1 mL of LB medium to an OD_600_ value of 1.5. A mixture of 160 µL of LB medium and 40 µL of the diluted overnight culture of *E. coli* BL21(DE3), 38 µL of the diluted overnight culture of *E. coli* BL21(DE3) and 2 µL of the diluted overnight culture of *S. aureus,* or 32 µL of the diluted overnight culture of *E. coli* BL21(DE3) and 8 µL of the diluted overnight culture of *S. agalactiae* was dispensed into 96-well plates. On the basis of the OD_600_ value of the *E. coli* BL21(DE3) culture, phage lysate was added to each well of a 96-well plate at an MOI of 0.001. The 96-well plates were placed into a TECAN Infinite 200 microplate reader, and the OD_600_ was measured every 15 min at 37°C with agitation.

### Luciferase assay

The OD_600_ value of the log-phase culture of *E. coli* BL21(DE3) was measured. T7 phage mutants harboring the *firefly* luciferase genes were inoculated into 1 mL of log-phase *E. coli* BL21(DE3) at an MOI of 0.0001 and cultured at 37°C with shaking at 950 rpm in an Allsheng Thermo Shaker incubator until the lysate became clear. Twenty microliters of lysates were used for the luciferase assay using the Single-Luciferase (Firefly) Reporter Assay Kit (TransDetect, cat. no. FR101-01) according to the manufacturer’s instructions.

### Measurement of phage one-step growth curves

The one-step growth experiment for the T7 wild-type and mutant phages was performed as previously described (47). Briefly, the OD_600_ value of the log-phase culture of *E. coli* BL21(DE3) was measured. One milliliter of log-phase *E. coli* BL21(DE3) was centrifuged at 6,100 × *g* for 2 min and resuspended in 1 mL of LB medium. Phages were added at an MOI of 0.0001, and the cultures were incubated at 37°C for 5 min for phage adsorption. Unadsorbed phages were removed from the cultures by centrifugation at 6,100 × *g* for 2 min. The pellets were resuspended in 9 mL of LB medium, and 10 µL of the culture was taken immediately for plaque-forming unit (PFU) counting. PFUs at this point (PFU_0_) were recognized as the number of infected *E. coli* cells. The cultures were incubated at 37°C with agitation. Ten microliters of the culture was taken every 10 min, serially diluted, and plated with *E. coli* BL21(DE3) to count PFUs. The values of PFUs per infected cell were calculated by dividing each PFU by PFU_0_.

### Lysin protein purification and detection

T7 phage mutants carrying the lysin genes were added to 50 mL of log-phase *E. coli* BL21(DE3) (OD_600_ ~1.0, 10^9^ cells mL^−1^) at an MOI of 0.0002 and incubated at 37°C at 200 rpm until the culture became clear. The cell debris was removed by centrifugation at 9,500 × *g* for 30 min at 4°C. The supernatant was applied to Ni-IDA Sepharose Resin (Sangon, cat. no. C600029). LysGH15 and LyJDKSS2 proteins were eluted with 250 mM imidazole in Tris-HCl buffer (25 mM Tris, 200 mM NaCl, 250 mM imidazole, pH 7.2). The protein mixture was concentrated with a 10 kDa MWCO Amicon Ultra Centrifugal Filter (Millipore, cat. no. UFC9010) and detected by SDS‒PAGE.

### Statistical analysis

Statistical analysis comparing two groups was performed using a two-sided Student’s *t* test. *P < 0.05; **P < 0.01; ***P < 0.001; ns, not significant. The error bars indicate the standard deviations.

## Data Availability

The plasmids and BAC constructs containing the clonable ΦX174-phage and T7-phage segments were deposited at Addgene. Their nucleic acid sequences have been uploaded to Addgene and NCBI. The genome sequences of the engineered T7-B, T7-B-G, T7-B-J, and T7-B-GJ phages have been uploaded to NCBI. The Addgene IDs and NCBI accession numbers are provided in Table S1.

## SUPPLEMENTAL MATERIAL

Supplemental material mbio.03582-24-s0001.pdf Supplemental figures and tables.

ASM does not own the copyrights to Supplemental Material that may be linked to, or accessed through, an article. The authors have granted ASM a non-exclusive, world-wide license to publish the Supplemental Material files. Please contact the corresponding author directly for reuse.
