# Supplementary material for: Programming virulent bacteriophages by developing a multiplex genome engineering method
Source: mBio. 2025 May 23;16(6):e03582-24. doi: 10.1128/mbio.03582-24 (PMC12153263; doi:10.1128/mbio.03582-24)
Supplement: Supplemental material — Supplemental figures and tables. [file mbio.03582-24-s0001.pdf]

Supplementary Information

Programming virulent bacteriophages by developing a multiplex genome engineering method

Hailin Zhang[1,2], Ru Zhu[1], Zhaofei Wang[3], Ruoting He[1], Yuran Zhang[2], Ji Luan[1], Yaxian Yan[3], Youming Zhang[1] & Hailong Wang[1,4,*]

[1]State Key Laboratory of Microbial Technology, Institute of Microbial Technology, Helmholtz International Lab for Anti-infectives, Shandong University–Helmholtz Institute of Biotechnology, Shandong University, Qingdao, Shandong, 266237, China

[2]School of Life Sciences, Jining Medical University, No. 669 Xueyuan Road, Donggang District, Rizhao, Shandong Province, 276826, China

[3]School of Agriculture and Biology, Shanghai Jiao Tong University, Shanghai Key Laboratory of Veterinary Biotechnology, Shanghai, China.

[4]Rizhao Research Institute of Shandong University, Rizhao, Shandong, 276800, China

Correspondence should be addressed to Hailong Wang. Email: wanghailong@sdu.edu.cn

Fig. S1 Restriction analysis of BACs from colonies obtained in cloning of the whole ΦX174 phage genome into the BAC vector.

Fig. S2 Restriction analysis of BACs or plasmids from colonies obtained in cloning of the 1,964-bp (F1) and 3,422-bp (F2) segments.

Fig. S3 PCR analysis of BACs containing T7 phage segments.

Fig. S4 PCR analysis of BACs containing S1-1, S1-2 and S1-3 T7 phage genomic segments.

Fig. S5 PCR analysis of BACs containing S3-1 and S3-2 T7 phage genomic segments.

Fig. S6 PCR analysis of BACs containing S6-1 and S6-2 T7 phage genomic segments.

Fig. S7 PCR analysis of BACs containing F8 and F9 T7 phage genomic segments.

Fig. S8 PCR analysis of deletions of nonessential genes in T7 mutated phages with genome reduction.

Fig. S9 The genetic stability of exogenous DNA fragments in recombinant phage genome.

Fig. S10 PCR analysis of BAC-T7-F8 containing lysin genes.

Fig. S11 Evaluation of lytic activity of synthetic T7 progeny phages.

Fig. S12 PCR amplification of lysin genes in the genome of synthetic T7 progeny phages.

Table S1. Accession numbers of BACs, plasmids, and synthetic phages in the NCBI database and the Addgene public repository.

Table S2. Nonessential gene list in T7 phage genome (Accession number NC_001604).

Table S3. Gene annotation list of T7 phage genome (Accession number NC_001604).

Table S4. Bacteria and phages used in this work.

Table S5. Oligonucleotides for cloning of phage genomes.

Table S6. Oligonucleotides for PCR amplification of the *amp-ccdB* cassettes.

TableS7. Oligonucleotides for replacing the *amp-ccdB* cassette in the second round of recombineering of the Red-ccdB method.

Table S8. Oligonucleotides for PCR amplification of *firefly* luciferase reporter gene.

Table S9. Oligonucleotides for preparation of the 3-kb, 5-kb, and 7-kb DNA fragments.

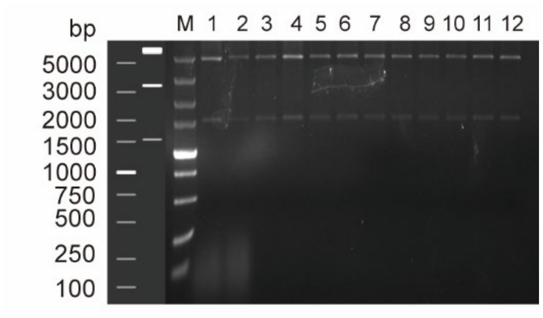

Fig. S1 Restriction analysis of BACs from colonies obtained in cloning of the whole ΦX174 phage genome into the BAC vector. The simulated gel for SacII+MscI restriction analysis of pBAC-ΦX174 is shown on the left panel. No correct clones were identified from 12 randomly selected colonies.

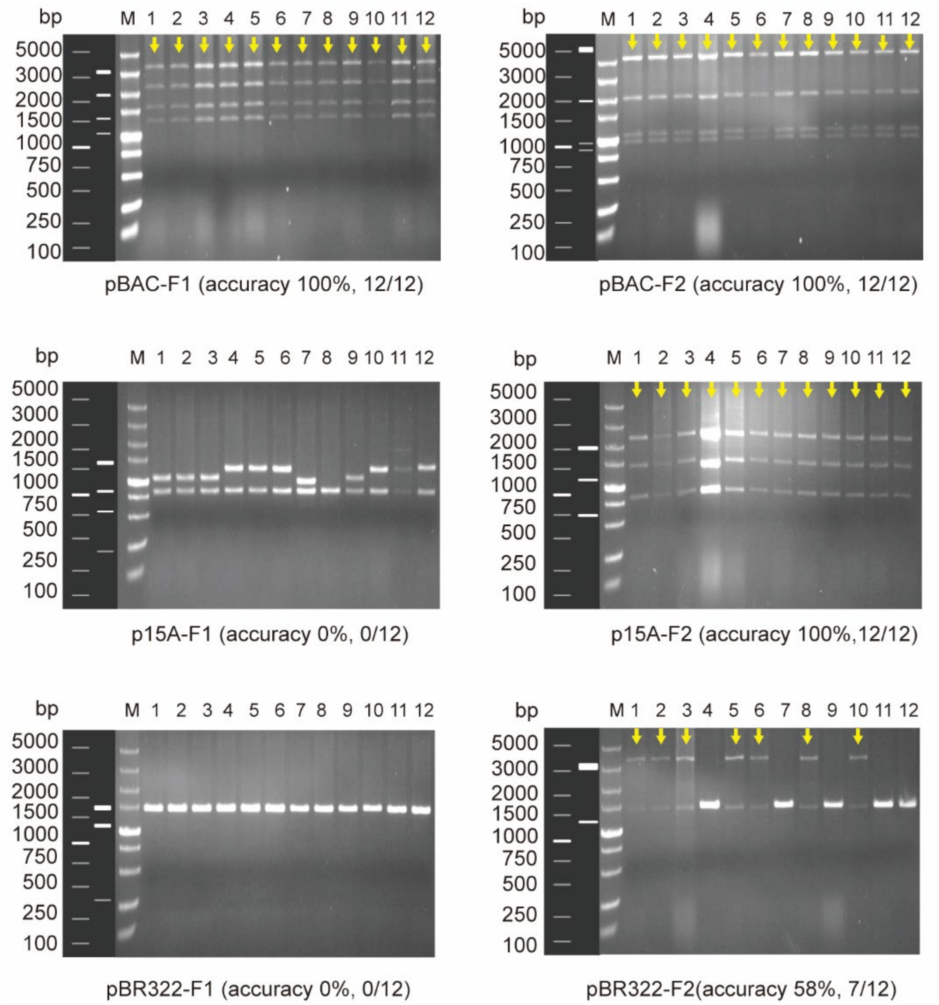

Fig. S2 Restriction analysis of BACs or plasmids from colonies obtained in cloning of the 1,964-bp (F1) and 3,422-bp (F2) segments. PstI+EcoRI was used for analysis of pBAC-F1 and pBR322-F1. PstI+SacII was used for analysis of pBAC-F2. EcoRV+XhoI was used for analysis of p15A-F1. SacII was used for analysis of p15A-F2. SacII+EcoRI was used for analysis of pBR322-F2. The simulated gel is shown on each left panel. Correct clones are indicated with arrows.

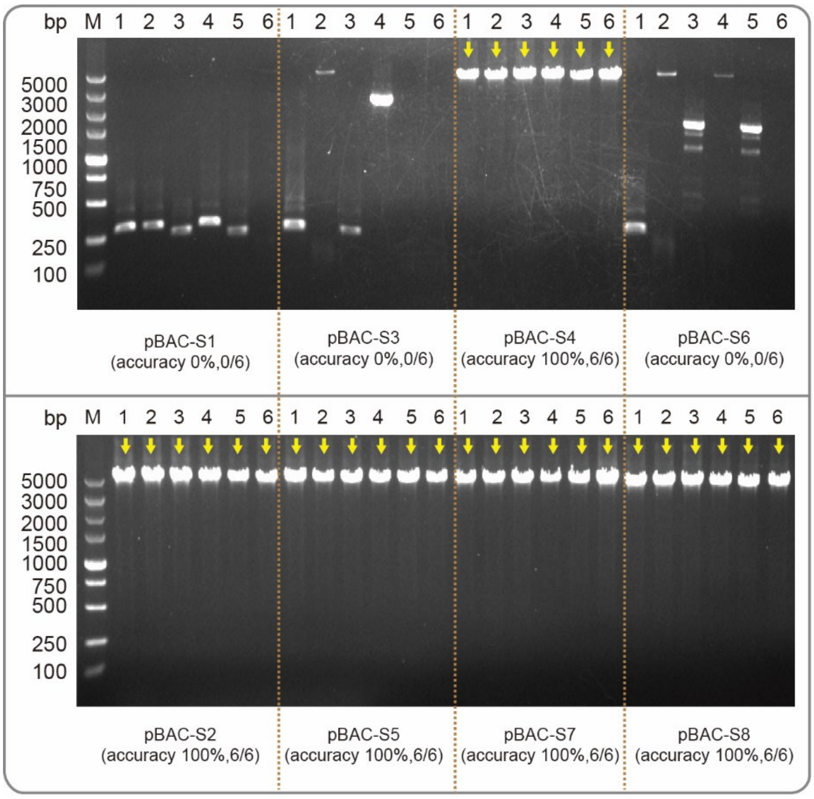

Fig. S3 PCR analysis of BACs containing T7 phage segments. Correct clones are indicated with arrows.

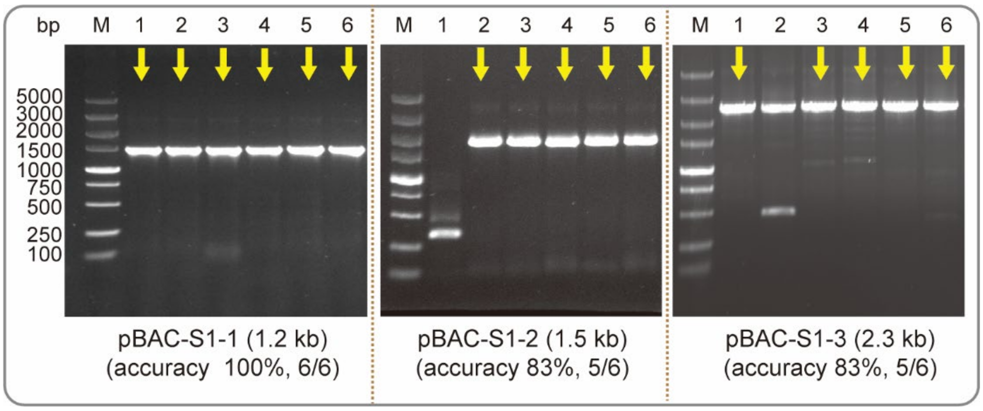

Fig. S4 PCR analysis of BACs containing S1-1, S1-2 and S1-3 T7 phage genomic segments. Correct clones are indicated with arrows.

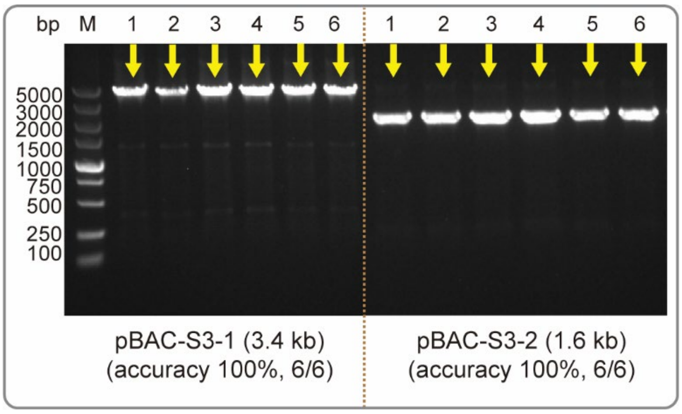

Fig. S5 PCR analysis of BACs containing S3-1 and S3-2 T7 phage genomic segments. Correct clones are indicated with arrows.

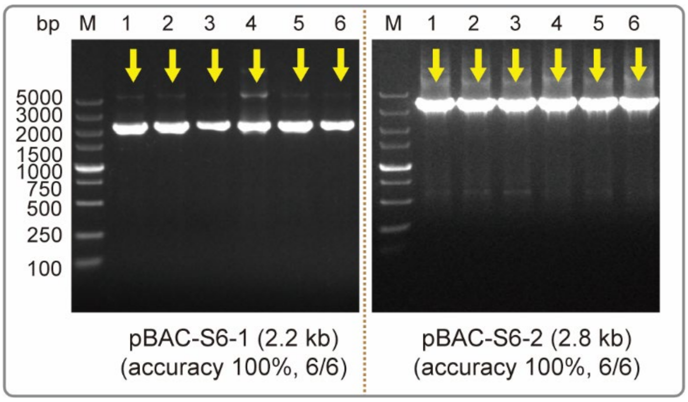

Fig. S6 PCR analysis of BACs containing S6-1 and S6-2 T7 phage genomic segments. Correct clones are indicated with arrows.

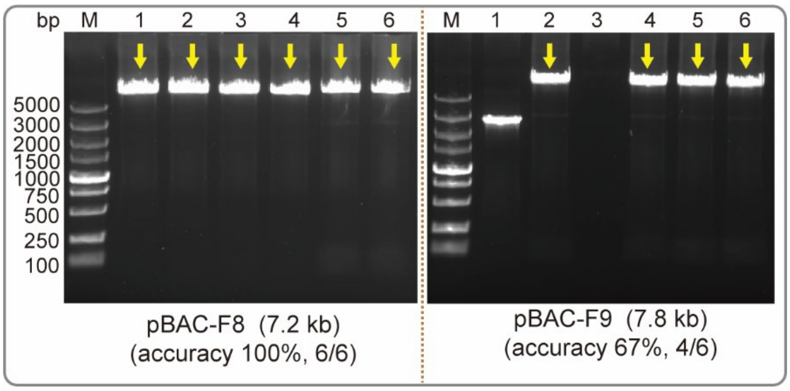

Fig. S7 PCR analysis of BACs containing F8 and F9 T7 phage genomic segments. Correct clones are indicated with arrows.

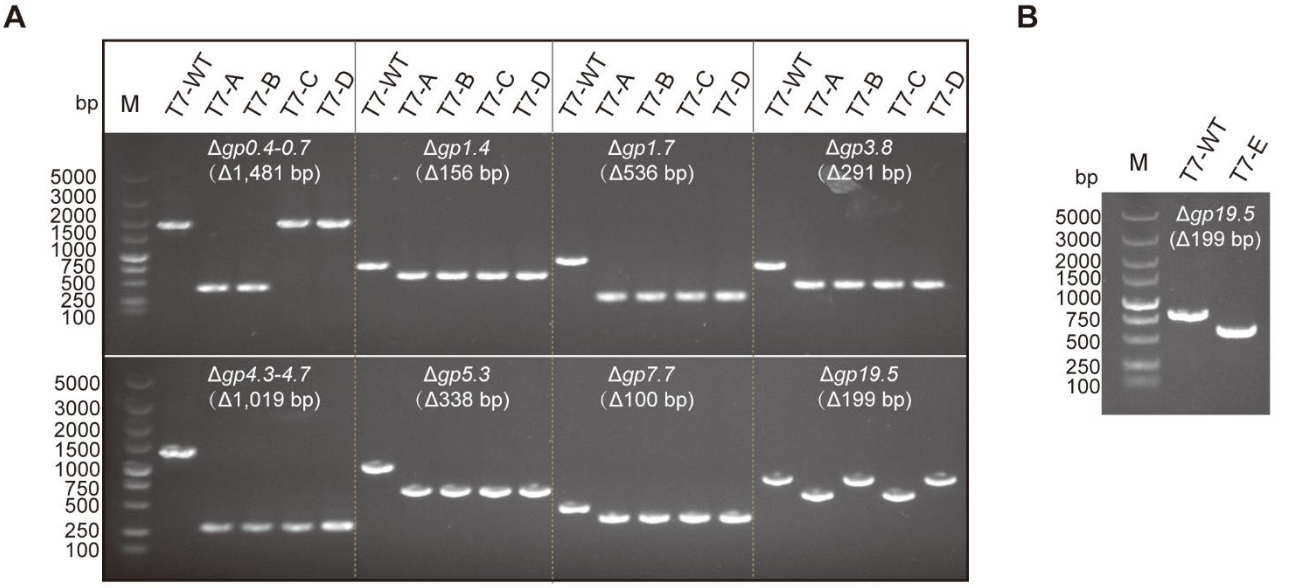

Fig. S8 PCR analysis of deletions of nonessential genes in T7 mutated phages with genome reduction. (A) PCR analysis of deletions of nonessential genes. (B) PCR analysis of deletion of *gp19.5* in T7-E genome.

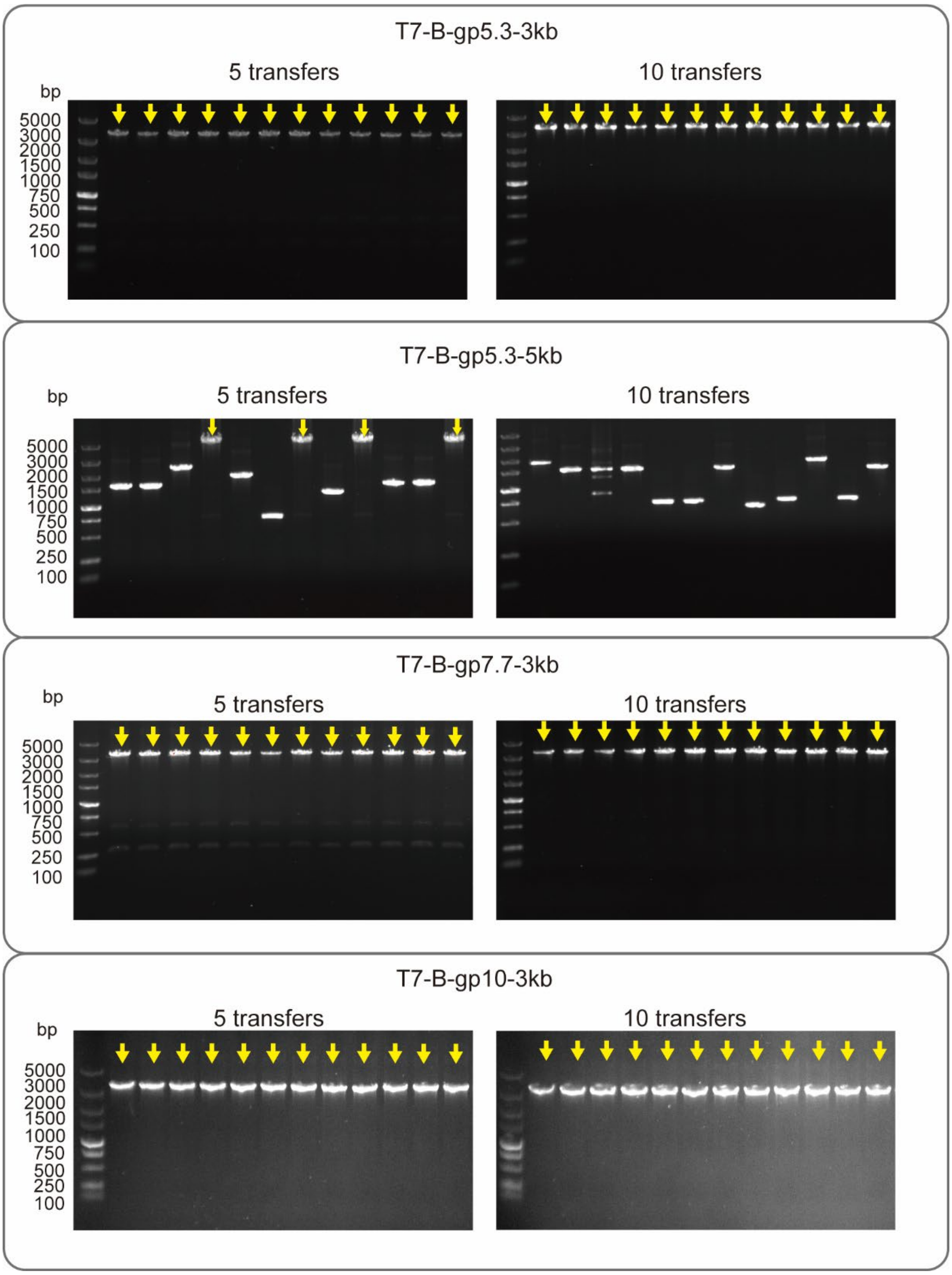

Fig. S9 The genetic stability of exogenous DNA fragments in recombinant phage genomes. All recombinant phages proliferated on *E. coli* BL21(DE3) at a MOI of 0.001. After 5 and 10 transfers, progeny phages were isolated and 12 phages of each recombinant phages were randomly selected respectively. The 3-kb or 5-kb exogenous DNA fragments were checked by PCR. Correct phages are indicated with arrows.

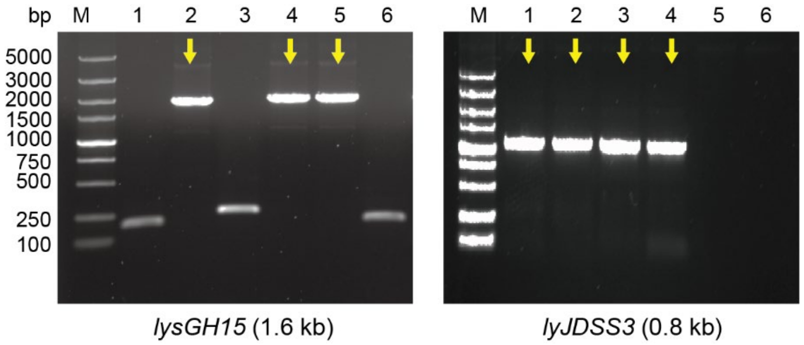

Fig. S10 PCR analysis of BAC-T7-F8 containing lysin genes. Correct clones are indicated with arrows.

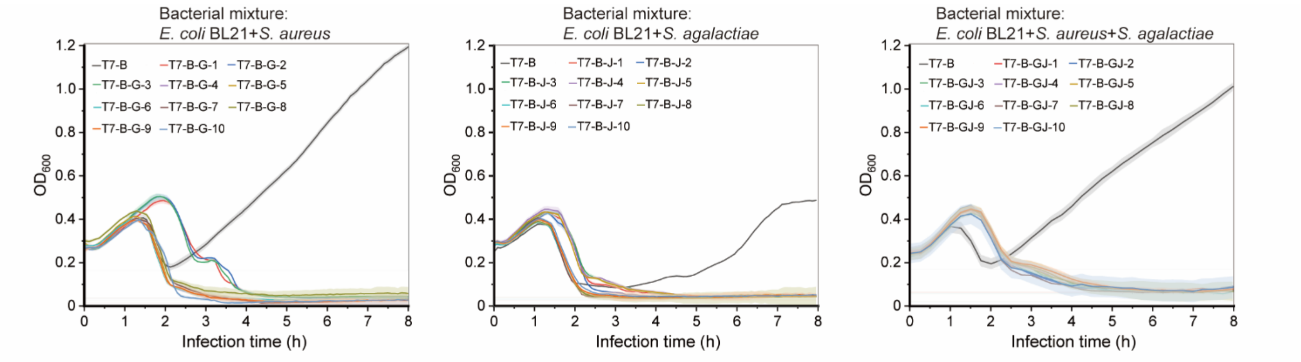

Fig. S11 Evaluation of lytic activity of synthetic T7 progeny phages. Ten progeny phages of T7-B-G, T7-B-J and T7-B-GJ were randomly selected after ten transfers and evaluated for lytic activity of respective target bacteria. Bacterial hosts and target bacteria are the same as T7-B-G, T7-B-J and T7-B-GJ (Fig 6). The lytic activity of T7-B was used as a control. OD_{600} value was measured every 15 min until 8 h. The data are presented as the means ± S.D.s. Each sample was performed in triplicate (*n* = 3). Error bars are shown as shaded error bar bands.

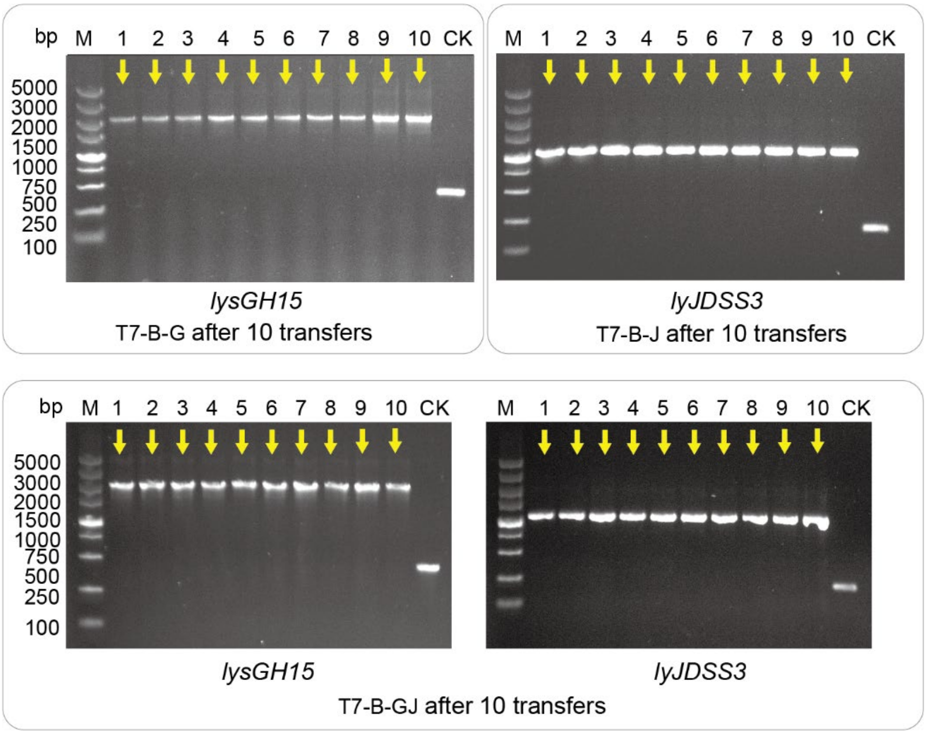

Fig. S12 PCR amplification of lysin genes in the genome of synthetic T7 progeny phages. Ten progeny phages of T7-B-G, T7-B-J and T7-B-GJ after 10 transfers were detected. The T7-B genomic DNA was used as a control (CK). Positive progeny phages are indicated with arrows.

Table S1 Accession numbers of BACs, plasmids, and synthetic phages in the NCBI database and the Addgene public repository.

Plasmids / Phages	NCBI accession numbers	Addgene IDs
pBAC-ΦX174-F1	PV223878	234977<br>https://www.addgene.org/234977/
p15A-ΦX174-F2	PV223879	234978<br>https://www.addgene.org/234978/
pBAC-T7-F1	PV173698	234967<br>https://www.addgene.org/234967/
pBAC-T7-F2	PV173699	234968<br>https://www.addgene.org/234968/
pBAC-T7-F3	PV223870	234969<br>https://www.addgene.org/234969/
pBAC-T7-F4	PV223871	234970<br>https://www.addgene.org/234970/
pBAC-T7-F5	PV223872	234971<br>https://www.addgene.org/234971/
pBAC-T7-F6	PV223874	234972<br>https://www.addgene.org/234972/
pBAC-T7-F7	PV223873	234973<br>https://www.addgene.org/234973/
pBAC-T7-F8	PV223875	234974<br>https://www.addgene.org/234974/
pBAC-T7-F9	PV223876	234975<br>https://www.addgene.org/234975/
pBAC-T7-F10	PV223877	234976<br>https://www.addgene.org/234976/
T7-B	PV223880	—
T7-B-G	PV223881	—
T7-B-J	PV223883	—
T7-B-GJ	PV223882	—

Table S2 Nonessential gene list in T7 phage genome (Accession number NC_001604) (1).

Nonessential gene regions	Start	End	Genes	size (bp)
gp0.4-0.7	1,257	2,737	*gp0.4, gp0.5, gp0.6A, gp0.6B, gp0.7A*	1,481
gp1.4	7,609	7,764	*gp1.4*	156
gp1.7	8,198	8,733	*gp1.7*	536
gp3.8	11,268	11,558	*gp3.8*	291
gp4.3-4.7	13,279	14,297	*gp4.3, gp4.5, gp4.7*	1,019
gp5.3	16,484	16,821	*gp5.3*	338
gp7.7	20,109	20,208	*gp7.7*	100
gp19.5	39,430	39,628	*gp19.5*	199

Table S3 Gene annotation list of T7 phage genome (Accession number NC_001604).

Gene	Start	End	Function	Classification
gp0.3	925	1,278	hypothetical protein	interaction with host
gp0.4	1,278	1,433	hypothetical protein	hypothetical gene
gp0.5	1,496	1,639	hypothetical protein	hypothetical gene
gp0.6	1,636	1,797	hypothetical protein	hypothetical gene
gp0.7	2,021	3,100	protein kinase	interaction with host
gp1.0	3,171	5,822	T3/T7-like RNA polymerase	DNA replication and nucleotide metabolism
gp1.1	6,007	6,135	hypothetical protein	hypothetical gene
gp1.3	6,475	7,554	ATP-dependent DNA ligase	nucleotide metabolism
gp1.4	7,608	7,763	hypothetical protein	hypothetical gene
gp1.5	7,791	7,880	hypothetical protein	hypothetical gene
gp1.7	8,166	8,756	hypothetical protein	hypothetical gene
gp1.8	8,749	8,895	hypothetical protein	hypothetical gene
gp2.0	8,898	9,092	inhibitor of host bacterial RNA polymerase	DNA replication and nucleotide metabolism
gp2.5	9,158	9,856	single-stranded DNA-binding protein	nucleotide metabolism
gp2.8	9,857	10,276	hypothetical protein	nucleotide metabolism
gp3.0	10,257	10,706	endonuclease I	DNA replication and nucleotide metabolism
gp3.5	10,706	11,161	lysozyme	nucleotide metabolism
gp3.8	11,225	11,590	putative NHN endonuclease	Nucleotide metabolism
gp4.0	11,565	13,265	DNA primase/helicase	DNA DNA replication and nucleotide metabolism
gp4.3	13,352	13,564	hypothetical protein	hypothetical gene
gp4.5	13,584	13,853	hypothetical protein	hypothetical gene
gp4.7	13,927	14,334	hypothetical protein	hypothetical gene
gp5.0	14,353	16,467	DNA polymerase	DNA replication and nucleotide metabolism
gp5.3	16,483	16,839	hypothetical protein	nucleotide metabolism
gp5.7	17,150	17,359	hypothetical protein	hypothetical gene
gp6.0	17,504	18,406	exonuclease	DNA replication and nucleotide metabolism

gp6.5	18,605	18,859	hypothetical protein	hypothetical gene
gp7.0	19,130	19,531	hypothetical protein	hypothetical gene
gp7.3	19,535	19,834	tail assembly protein	structural gene
gp7.7	19,848	20,240	hypothetical protein	nucleotide metabolism
gp8.0	20,240	21,850	head-tail connector protein	structural gene
gp9.0	21,950	22,873	capsid assembly protein	structural gene
gp10.0	22,967	23,989	major capsid protein	structural gene
gp11.0	24,228	24,818	tail tubular protein A	structural gene
gp12.0	24,842	27,226	tail tubular protein B	structural gene
gp13.0	27,307	27,723	internal virion protein A	structural gene
gp14.0	27,728	28,318	internal virion protein B	structural gene
gp15.0	28,325	30,568	internal virion protein C	structural gene
gp16.0	30,595	34,551	internal virion protein D	structural gene
gp17.0	34,624	36,285	tail fiber protein	structural gene
gp17.5	36,344	36,547	type II holin	lysis
gp18.0	36,553	36,822	DNA packaging protein, small subunit	packaging
gp18.5	36,917	37,348	phage lambda Rz-like lysis protein	lysis
gp19.2	38,016	38,273	hypothetical protein	hypothetical gene
gp19.3	38,553	38,726	hypothetical protein	hypothetical gene
gp19.5	39,389	39,538	hypothetical protein	hypothetical gene

Table S4 Bacteria and phages used in this work.

**Strains**	**Genotype or relevant features**
E. coli GB2005 (2)	(HS996, Δ*recET*, Δ*ybcC*), The endougenous *recET* locus and DLP12 prophage *ybcC*, which encodes a putative exonuclease, were deleted
E. coli GB05-red (2)	(GB2005, araC-BAD-αβγA) lambda *red* operon and *recA* under P_{BAD} promoter were inserted at the *ybcC* locus
E. coli GB05-dir (3)	(GB2005, araC-BAD-ETgA) *recE, recT* and *recA* under P_{BAD} promoter were inserted at the *ybcC* locus
E. coli GBred-gyrA462 (2)	GB05-red, which carries the arabinose inducible *redγβαA* operon integrated at *ybcC*, was mutated with an oligonucleotide directed to change the Arg462 codon of gyrase into the cysteine codon TGC
S. aureus	*Staphylococcus aureus* ATCC 25923
S. agalactiae	*Streptococcus agalactiae* ATCC13813
ΦX174 phage	Accession No. CP004084
T7 phage	Accession No. NC_001604

Table S5 Oligonucleotides for cloning of phage genomes.

Name	sequence (5'-3')	Template	Purpose
pBAC-ΦX174-E-F	GGCTCACCTTCACGGGTGGGCCTTTCTTCG***GTATAC***AAAGCCGCTGAATTGTTCGC	Genomic DNA of ΦX174 phage	Construction of pBAC-ΦX174
pBAC-ΦX174-E-R	ACGGCTCACCTTCGGGTGGGCCTTTCTGCG***GTATAC***AACCGGACGCTCGACGCCAT		
pBAC-ΦX174-F1-F	GGCTCACCTTCACGGGTGGGCCTTTCTTCG***GTATAC***TTTTGACTTTGAGCGTATCG		Splitting the phage genome to obtain the F1 segment
pBAC-ΦX174-F1-R	ACGGCTCACCTTCGGGTGGGCCTTTCTGCG***GTATAC***GTAAACGCGAACAATTCAGC		
pBAC-ΦX174-F2-F	GGCTCACCTTCACGGGTGGGCCTTTCTTCG***GTATACC***ATGGAAGGCGCTGAATTTAC		Splitting the phage genome to obtain the F2 segment
pBAC-ΦX174-F2-R	ACGGCTCACCTTCGGGTGGGCCTTTCTGCG***GTATAC***AGCATTGGGGATTGAGAAAG		
pBAC-T7Te-F	CGCAGAAAGGCCCACCCGAAGGTGAGCCGTTTAAGGGCACCAATAACT	pBAC-cm-BR322-ccdB-hyg (4)	Amplification of the pBAC vector for construction of recombinant plasmids
pBAC-T3Te-R	CGAAGAAAGGCCCACCCGTGAAGGTGAGCCACAGGTTACGACGACATGTC		
p15A-T7Te-F	CGCAGAAAGGCCCACCCGAAGGTGAGCCCCTAGGGATATATTCCGCTTC	p15A-cm-ccdB (2)	Amplification of the p15A vector for construction of recombinant plasmids
p15A-T3Te-R	CGAAGAAAGGCCCACCCGTGAAGGTGAGCCTATTCGGCCTTGAATTGATC		
pBR322-T7Te-F	CGCAGAAAGGCCCACCCGAAGGTGAGCCACAAATGGCAAGGGCTAATG	pBR322-cm-ccdB-rpsl (4)	Amplification of the pBR322 vector for construction of recombinant plasmids
pBR322-T3Te-R	CGAAGAAAGGCCCACCCGTGAAGGTGAGCCTGATCGGCACGTAAGAGGTT		
pBAC-T7-S1-1-F	GGCTCACCTTCACGGGTGGGCCTTTCTTCG***GCCGGC***TCTCACAGTGTACGGACCTA	Genomic DNA of T7 phage	Splitting T7 genome and construction of BACs carrying T7 genome segments (S1-S8).
pBAC-T7-S1-1-R	GGCTCACCTTCGGGTGGGCCTTTCTGCG***GCCGGC***TCCTCGACTTCCTCCAAGTA		
pBAC-T7-S1-2-F	GGCTCACCTTCACGGGTGGGCCTTTCTTCG***GCCGGC***CAGAAGACTTGCTCAATGAA		
pBAC-T7-S1-2-R	GGCTCACCTTCGGGTGGGCCTTTCTGCG***GCCGGC***TTCCTCGACTTCCTTGATGA		
pBAC-T7-S1-3-F	GGCTCACCTTCACGGGTGGGCCTTTCTTC***GGCCGGC***CAGAAGACTTGCTCAATGAATACT TGGAGGAAGTCGAGGAGTCGCACGACAGAAAGAAAT		
pBAC-T7-S1-3-R	GGCTCACCTTCGGGTGGGCCTTTCTGCG***GCCGGC***ACTGCGAGTAACACCGTAAG		

pBAC-T7-S2-F	GGCTCACCTTCACGGGTGGGCCTTTCTTCG***GCCGGC***AAGTCAAGCTGGGCACTAAG		
pBAC-T7-S2-R	GGCTCACCTTCGGGTGGGCCTTTCTGCG***GCCGGC***ATGGATTATCACAGGAGTGC		
pBAC-T7-S3-1-F	GGCTCACCTTCACGGGTGGGCCTTTCTTCG***GCCGGC***GTCTAATGCTCCGAAAGGTT		
pBAC-T7-S3-1-R	GGCTCACCTTCGGGTGGGCCTTTCTGCG***GCCGGC***TGTCAGCCAGTTCGTTGGAG		
pBAC-T7-S3-2-F	GGCTCACCTTCACGGGTGGGCCTTTCTTCG***GCCGGC***TAAAGAGGCCACACAGTCAC		
pBAC-T7-S3-2-R	GGCTCACCTTCGGGTGGGCCTTTCTGCG***GCCGGC***TTGCTTTTGTGTCAAACGGG		
pBAC-T7-S4-F	GGCTCACCTTCACGGGTGGGCCTTTCTTCG***GCCGGC***ATGGCTGCTCGCTAAACAAG		
pBAC-T7-S4-R	GGCTCACCTTCGGGTGGGCCTTTCTGCG***GCCGGC***CCTGTCTACCTTTGGTAACC		
pBAC-T7-S5-F	GGCTCACCTTCACGGGTGGGCCTTTCTTCG***GCCGGC***GAACTCCAAAGGATAATTCC		
pBAC-T7-S5-R	GGCTCACCTTCGGGTGGGCCTTTCTGCG***GCCGGC***ACCAGAAAGGTCGAACACTC		
pBAC-T7-S6-1-F	GGCTCACCTTCACGGGTGGGCCTTTCTTCG***GCCGGC***TATTACGCTGTGTTCACTGG		
pBAC-T7-S6-1-R	GGCTCACCTTCGGGTGGGCCTTTCTGCG***GCCGGC***CAGCCTTAGCTTCAAGAATG		
pBAC-T7-S6-2-F	GGCTCACCTTCACGGGTGGGCCTTTCTTCG***GCCGGC***GACTTTGAGGTATTCACTCC		
pBAC-T7-S6-2-R	GGCTCACCTTCGGGTGGGCCTTTCTGCG***GCCGGC***ATCACCAGTGAACGTGTAGG		
pBAC-T7-S7-F	GGCTCACCTTCACGGGTGGGCCTTTCTTCG***GCCGGC***TGGAGCAGATGACCAAGTTC		
pBAC-T7-S7-R	GGCTCACCTTCGGGTGGGCCTTTCTGCG***GCCGGC***CTCCGCTTGGTTTCTGAAAG		
pBAC-T7-S8-F	GGCTCACCTTCACGGGTGGGCCTTTCTTCG***GCCGGC***AATGAAGCCTTACAGTTCCG		
pBAC-T7-S8-R	GGCTCACCTTCGGGTGGGCCTTTCTGCG***GCCGGC***AGGGACACAGAGAGACACTC		

Underlined sequences are homology arms for cloning using the RecET method. Italic and bold letters are BstZ17I or NaeI restriction sites.

Table S6 Oligonucleotides for PCR amplification of the *amp-ccdB* cassettes.

**Name**	**sequence (5'-3')**	**Template**
Amp-ccdB-gp1.4-F	CTTCGGGTGGGCCTTTCTGCGTTTATAAGGAGACACTTTATCAACAGGTTGAACTTTTG	p15A-amp-ccdB (2)
Amp-ccdB-gp1.4-R	ATTAAGTACATCATGGTGTGTACCTCCTTTAGTGAGTCGTTTTGTTTATTTTTCTAAATAC	
Amp-ccdB-gp1.7-F	GTGTTCTGATGGGACTGTTAGATGGTGAAGCCTGGGAAAATCAACAGGTTGAACTTTTG	
Amp-ccdB-gp1.7-R	GTGGGGTTGACTTGAAGTTATGCATAACATTTATCCTTATTTTGTTTATTTTTCTAAATAC	
Amp-ccdB-gp3.8-F	CGCAAGTCTTATAAACAATTCTATAAGGCTCCGAGGAGGCTCAACAGGTTGAACTTTTG	
Amp-ccdB-gp3.8-R	GAAATACACTATCGGAATCGTGCGAATTGTCCATGCAATTTTTGTTTATTTTTCTAAATAC	
Amp-ccdB-gp4.3-F	GACTGGTCCAACGACACTGACTTCTGACAGGATTCTTGATTCAACAGGTTGAACTTTTG	
Amp-ccdB-gp4.3-R	TGATTATCGTGACTTAACAATCTCTTCATATGAAACAACTTTTGTTTATTTTTCTAAATAC	
Amp-ccdB-gp5.3-F	CCTAATTGGGCGATTTGCCACTGATACAGGAGGCTACTCATCAACAGGTTGAACTTTTG	
Amp-ccdB-gp5.3-R	GTCATAGCCATAATAATTTCTCCTATAGTTTTATGCCTTTTTTGTTTATTTTTCTAAATAC	
Amp-ccdB-gp7.7-F	TACCAAAGGTAGACAGGCTAAAGGAGAGGAACTAAGCAAGTCAACAGGTTGAACTTTTG	
Amp-ccdB-gp7.7-R	CTCAGCCATTAAATGTGTCTCCATGTCTTACGCTGTAGTATTTGTTTATTTTTCTAAATAC	
Amp-ccdB-gp19.5-F	TGTTCCGCTTATTGTTGAACCTACTGCGGCATAGAGTCACTCAACAGGTTGAACTTTTG	
Amp-ccdB-gp19.5-R	TAGACCTTAGGATAGACCATTAGAGGCTCTTTATGGGTATTTTGTTTATTTTTCTAAATAC	

Underlined sequences are homology arms for recombineering.

Table S7 Oligonucleotides for replacing the *amp-ccdB* cassette in the second round of recombineering of the Red-ccdB method.

**Name**	**sequence (5'-3')**
HA-gp1.4	CTTCGGGTGGGCCTTTCTGCGTTTATAAGGAGACACTTTACTTCGGGTGGGCCTTTCTGCGTTTATAAGGAGACACTTTA
HA-gp1.7	GTGTTCTGATGGGACTGTTAGATGGTGAAGCCTGGGAAAAATAAGGATAAATGTTATGCATAACTTCAAGTCAACCCCAC
HA-gp3.8	CGCAAGTCTTATAAACAATTCTATAAGGCTCCGAGGAGGCAATTGCATGGACAATTCGCACGATTCCGATAGTGTATTTC
HA-gp4.3	GACTGGTCCAACGACACTGACTTCTGACAGGATTCTTGATAGTTGTTTCATATGAAGAGATTGTTAAGTCACGATAATCA
HA-gp5.3	CCTAATTGGGCGATTTGCCACTGATACAGGAGGCTACTCAAAAGGCATAAAACTATAGGAGAAATTATTATGGCTATGAC
HA-gp7.7	TACCAAAGGTAGACAGGCTAAAGGAGAGGAACTAAGCAAGTACTACAGCGTAAGACATGGAGACACATTTAATGGCTGAG
HA-gp19.5	TGTTCCGCTTATTGTTGAACCTACTGCGGCATAGAGTCACATACCCATAAAGAGCCTCTAATGGTCTATCCTAAGGTCTA

Underlined sequences are homology arms for recombineering.

Table S8 Oligonucleotides for PCR amplification of *firefly* luciferase reporter gene.

Name	sequence (5'-3')	Template
gp0.4-PJ23119-F	CAGAAGACTTGCTCAATGAATACTTGGAGGAAGTCGAGGATTGACAGCTAGCTCAGTCC	p15A-cm-J23119-firefly or p15A-cm-T7-firefly (constructed in this study)
gp0.4-PJ23119-R	TACGGGCCTTAGCGCGGTCAATTTCTTTCTGTCGTGCGACTTACAATTTGGACTTTCCG	
gp0.4-PT7-F	CAGAAGACTTGCTCAATGAATACTTGGAGGAAGTCGAGGATAATACGACTCACTATAGG	
gp0.4-PT7-R	TACGGGCCTTAGCGCGGTCAATTTCTTTCTGTCGTGCGACTTACAATTTGGACTTTCCG	
gp1.4-PJ23119-F	CTTCGGGTGGGCCTTTCTGCGTTTATAAGGAGACACTTTATTGACAGCTAGCTCAGTCC	
gp1.4-PJ23119-R	ATTAAGTACATCATGGTGTGTACCTCCTTTAGTGAGTCGTTTACAATTTGGACTTTCCG	
gp1.4-PT7-F	CTTCGGGTGGGCCTTTCTGCGTTTATAAGGAGACACTTTATAATACGACTCACTATAGG	
gp1.4-PT7-R	ATTAAGTACATCATGGTGTGTACCTCCTTTAGTGAGTCGTTTACAATTTGGACTTTCCG	
gp1.7-PJ23119-F	GTGTTCTGATGGGACTGTTAGATGGTGAAGCCTGGGAAAATTGACAGCTAGCTCAGTCC	
gp1.7-PJ23119-R	GTGGGGTTGACTTGAAGTTATGCATAACATTTATCCTTATTTACAATTTGGACTTTCCG	
gp1.7-PT7-F	GTGTTCTGATGGGACTGTTAGATGGTGAAGCCTGGGAAAATAATACGACTCACTATAGG	
gp1.7-PT7-R	GTGGGGTTGACTTGAAGTTATGCATAACATTTATCCTTATTTACAATTTGGACTTTCCG	
gp3.8-PJ23119-F	CGCAAGTCTTATAAACAATTCTATAAGGCTCCGAGGAGGCTTGACAGCTAGCTCAGTCC	
gp3.8-PJ23119-R	GAAATACACTATCGGAATCGTGCGAATTGTCCATGCAATTTTACAATTTGGACTTTCCGC	
gp3.8-PT7-F	CGCAAGTCTTATAAACAATTCTATAAGGCTCCGAGGAGGCTAATACGACTCACTATAGG	
gp3.8-PT7-R	GAAATACACTATCGGAATCGTGCGAATTGTCCATGCAATTTTACAATTTGGACTTTCCGC	
gp4.3-PJ23119-F	GACTGGTCCAACGACACTGACTTCTGACAGGATTCTTGATTTGACAGCTAGCTCAGTCC	
gp4.3-PJ23119-R	TGATTATCGTGACTTAACAATCTCTTCATATGAAACAACTTTACAATTTGGACTTTCCGC	

gp4.3-PT7-F	GACTGGTCCAACGACACTGACTTCTGACAGGATTCTTGATTAATACGACTCACTATAGG
gp4.3-PT7-R	TGATTATCGTGACTTAACAATCTCTTCATATGAAACAACTTTACAATTTGGACTTTCCGC
gp5.3-PJ23119-F	CCTAATTGGGCGATTTGCCACTGATACAGGAGGCTACTCATTGACAGCTAGCTCAGTCC
gp5.3-PJ23119-R	GTCATAGCCATAATAATTTCTCCTATAGTTTTATGCCTTTTTACAATTTGGACTTTCCGC
gp5.3-PT7-F	CCTAATTGGGCGATTTGCCACTGATACAGGAGGCTACTCATAATACGACTCACTATAGG
gp5.3-PT7-R	GTCATAGCCATAATAATTTCTCCTATAGTTTTATGCCTTTTTACAATTTGGACTTTCCGC
gp7.7-PJ23119-F	TACCAAAGGTAGACAGGCTAAAGGAGAGGAACTAAGCAAGTTGACAGCTAGCTCAGTCC
gp7.7-PJ23119-R	CTCAGCCATTAAATGTGTCTCCATGTCTTACGCTGTAGTATTACAATTTGGACTTTCCGC
gp7.7-PT7-F	TACCAAAGGTAGACAGGCTAAAGGAGAGGAACTAAGCAAGTAATACGACTCACTATAGG
gp7.7-PT7-R	CTCAGCCATTAAATGTGTCTCCATGTCTTACGCTGTAGTATTACAATTTGGACTTTCCGC
gp19.5-PJ23119-F	TGTTCCGCTTATTGTTGAACCTACTGCGGCATAGAGTCACTTGACAGCTAGCTCAGTCC
gp19.5-PJ23119-R	TAGACCTTAGGATAGACCATTAGAGGCTCTTTATGGGTATTTACAATTTGGACTTTCCGC
gp19.5-PT7-F	TGTTCCGCTTATTGTTGAACCTACTGCGGCATAGAGTCACTAATACGACTCACTATAGG
gp19.5-PT7-R	TAGACCTTAGGATAGACCATTAGAGGCTCTTTATGGGTATTTACAATTTGGACTTTCCGC

Underlined sequences are homology arms for recombineering.

Table S9 Oligonucleotides for preparation of the 3-kb, 5-kb, and 7-kb DNA fragments.

Name	sequence (5'-3')	Template
gp5.3-3k-F	CCTAATTGGGCGATTTGCCACTGATACAGGAGGCTACTCATTACAATTTGGACTTTCCG	pBAC-cm-3k, pBAC-cm-5k, or pBAC-cm-7k (constructed in this study)
gp5.3-3k-R	GTCATAGCCATAATAATTTCTCCTATAGTTTTATGCCTTTGGTACAGTAGACAGTCCTT	
gp5.3-5k-F	CCTAATTGGGCGATTTGCCACTGATACAGGAGGCTACTCATTACAATTTGGACTTTCCG	
gp5.3-5k-R	GTCATAGCCATAATAATTTCTCCTATAGTTTTATGCCTTTCAAGATGGATTGCACGCAG	
gp5.3-7k-F	CCTAATTGGGCGATTTGCCACTGATACAGGAGGCTACTCATTACAATTTGGACTTTCCG	
gp5.3-7k-R	GTCATAGCCATAATAATTTCTCCTATAGTTTTATGCCTTTAGTATTCAACATTTCCGTG	
gp7.7-3k-F	TACCAAAGGTAGACAGGCTAAAGGAGAGGAACTAAGCAAGTTACAATTTGGACTTTCCG	
gp7.7-3k-R	CTCAGCCATTAAATGTGTCTCCATGTCTTACGCTGTAGTAGGTACAGTAGACAGTCCTT	
gp7.7-5k-F	TACCAAAGGTAGACAGGCTAAAGGAGAGGAACTAAGCAAGTTACAATTTGGACTTTCCG	
gp7.7-5k-R	CTCAGCCATTAAATGTGTCTCCATGTCTTACGCTGTAGTACAAGATGGATTGCACGCAG	
gp7.7-7k-F	TACCAAAGGTAGACAGGCTAAAGGAGAGGAACTAAGCAAGTTACAATTTGGACTTTCCG	
gp7.7-7k-R	CTCAGCCATTAAATGTGTCTCCATGTCTTACGCTGTAGTAAGTATTCAACATTTCCGTG	
gp10-3k-F	CCCAGAAGCTGCTGGTGCAGTGGTTTTCAAAGTGGAGTAATTACAATTTGGACTTTCCG	
gp10-3k-R	TCTTCGGGACTAGCAGCGACCGTTGAGGCCACCCCCAGCAGGTACAGTAGACAGTCCTT	
gp10-5k-F	CCCAGAAGCTGCTGGTGCAGTGGTTTTCAAAGTGGAGTAATTACAATTTGGACTTTCCG	
gp10-5k-R	TCTTCGGGACTAGCAGCGACCGTTGAGGCCACCCCCAGCACAAGATGGATTGCACGCAG	
gp10-7k-F	CCCAGAAGCTGCTGGTGCAGTGGTTTTCAAAGTGGAGTAATTACAATTTGGACTTTCCG	
gp10-7k-R	TCTTCGGGACTAGCAGCGACCGTTGAGGCCACCCCCAGCAAGTATTCAACATTTCCGTG	

Underlined sequences are homology arms for recombineering.

Supplementary references

1. Yuan S, Shi J, Jiang J, Ma Y. 2022. Genome-scale top-down strategy to generate viable genome-reduced phages. Nucleic Acids Res 50:13183-13197.
2. Wang H, Bian X, Xia L, Ding X, Müller R, Zhang Y, Fu J, Stewart AF. 2014. Improved seamless mutagenesis by recombineering using *ccdB* for counterselection. Nucleic Acids Res 42:e37.
3. Fu J, Bian X, Hu S, Wang H, Huang F, Seibert PM, Plaza A, Xia L, Müller R, Stewart AF, Zhang Y. 2012. Full-length RecE enhances linear-linear homologous recombination and facilitates direct cloning for bioprospecting. Nat Biotechnol 30:440-6.
4. Wang H, Li Z, Jia R, Hou Y, Yin J, Bian X, Li A, Muller R, Stewart AF, Fu J, Zhang Y. 2016. RecET direct cloning and Redαβ recombineering of biosynthetic gene clusters, large operons or single genes for heterologous expression. Nat Protoc 11:1175-90.
